# Integrin Mechano-chemical Signaling Generates Plasma Membrane Nanodomains that Promote Cell Spreading

**DOI:** 10.1016/j.cell.2019.04.037

**Published:** 2019-05-16

**Authors:** Joseph Mathew Kalappurakkal, Anupama Ambika Anilkumar, Chandrima Patra, Thomas S. van Zanten, Michael P. Sheetz, Satyajit Mayor

**Affiliations:** 1 National Centre for Biological Sciences, Tata Institute of Fundamental Research, Bellary Road, Bangalore, India; 2 Institute for Stem Cell Biology and Regenerative Medicine, Bellary Road, Bangalore, India; 3 Mechanobiology Institute, National University of Singapore, Singapore, Singapore; 4 St. Johns Research Institute, Bangalore, India

## Abstract

Glycosylphosphatidylinositol-anchored proteins (GPI-APs) are a major class of lipid-anchored plasma membrane proteins. GPI-APs form nanoclusters generated by cortical acto-myosin activity. While our understanding of the physical principles governing this process is emerging, the molecular machinery and functional relevance of GPI-AP nanoclustering are unknown. Here, we first show that a membrane receptor signaling pathway directs nanocluster formation. Arg-Gly-Asp motif-containing ligands bound to the β1-integrin receptor activate src and focal adhesion kinases, resulting in RhoA signaling. This cascade triggers actin-nucleation via specific formins, which, along with myosin activity, drive the nanoclustering of membrane proteins with actin-binding domains. Concurrently, talinmediated activation of the mechano-transducer vinculin is required for the coupling of the acto-myosin machinery to inner-leaflet lipids, thereby generating GPI-AP nanoclusters. Second, we show that these nanoclusters are functional; disruption of their formation either in GPI-anchor remodeling mutants or in vinculin mutants impairs cell spreading and migration, hallmarks of integrin function.

## Introduction

Proteins and lipids can laterally segregate along the plasma membrane (PM) into domains that play a pivotal role in the spatio-temporal regulation of many cellular processes. Such functional domains, enriched in cholesterol, sphingolipids, and outer-leaflet lipid-tethered glycosylphosphatidylinositol-anchored proteins (GPI-APs), have often been termed as membrane rafts (Lingwood and Simons, 2010). Cellular processes including T cell activation (Gaus et al., 2005), B cell receptor activation (Gupta and DeFranco, 2007), and cell adhesion (Gaus et al., 2006; van Zanten et al., 2009) are accompanied by the generation of membrane domains. How membrane domains form remains controversial. Features of membrane domains, like their size and dynamics, are very different in cells, when compared to domains observed in artificial membranes and cell-free membrane preparations, that result from large-scale phase segregation processes (Sezgin et al., 2012). In cells, many of the raft-enriched components such as outer-leaflet GPI-APs, gangliosides, and inner-leaflet Ras proteins form nanoclusters at the PM (Fujita et al., 2007; Prior et al., 2003; Varma and Mayor, 1998). We had previously proposed that nanoclusters of GPI-APs are driven by transient remodeling contractile platforms at the inner leaflet called “asters,” composed of dynamic actin filaments and myosin motors (Gowrishankar et al., 2012). These asters immobilize long-acyl-chain-containing phosphatidylserine (PS) at the inner leaflet. PS interacts across the bilayer with long-acyl-chain-containing GPI-APs at the outer leaflet to facilitate GPI-AP nanoclustering (Raghupathy et al., 2015).

Theoretical work (Gowrishankar et al., 2012; Husain and Rao, 2017) together with reconstitution studies (Köster et al., 2016) indicates that membranes are active actin-membrane composites (Rao and Mayor, 2014). In this context, membrane components can be classified as *inert*, *passive*, and *active*, based on their ability to couple with and regulate the actin machinery (Gowrishankar et al., 2012). Inert molecules cannot interact with actin, passive molecules can interact with actin filaments, and active molecules can interact with actin and influence local actin dynamics. In doing so, active components regulate their own organization and those of passive molecules in their vicinity.

Upon binding of the ligand intercellular adhesion molecule 1 (ICAM-1) to its cognate integrin receptor, lymphocyte function-associated antigen-1 (LFA-1), in immune cells, the fraction of GPI-APs in nanoclusters increases from ~30% to ~80% near LFA-1. Reduction of cholesterol levels inhibits the formation of GPI-AP nanoclusters (Sharma et al., 2004) and concurrently reduces the ligand binding capacity of the LFA-1 receptors (van Zanten et al., 2009). Nanoclusters can concentrate into larger-scale mesoscopic domains or “hotspots” (Goswami et al., 2008; van Zanten et al., 2009). Regulation of nanoclusters may be important for signal transduction processes at the cell surface (Chaudhuri et al., 2011; Tian et al., 2007).

While our understanding of nanocluster formation is growing, we still do not know what nucleates actin, what triggers myosin function, or how actin is connected to PS lipids. In this manuscript, we provide some insights into these questions. We find that upon engagement with Arg-Gly-Asp (RGD)-containing ligands, integrin receptors activate focal adhesion kinase (FAK) and src family kinases (SFK) culminating in RhoA triggered formin activity, necessary for the generation of the dynamic actin filaments. RhoA also activates the ROCK pathway, required for myosin activation. Thus, integrin receptors as such, are *active* species, which upon ligand binding generates the actin machinery that builds clusters at the PM. Additionally, we identify vinculin, a ubiquitous protein that associates with integrins in focal adhesions (FAs) (Atherton et al., 2016), that, upon mechano-sensitive activation, couples the integrin-dependent signaling pathway to the generation of GPI-AP nanoclusters. Furthermore, using GPI-anchor remodeling mutants as well as vinculin mutants that fail to support nanocluster formation, we show that the nanoclusters created by this active machinery are essential for integrin-mediated cell spreading and migration. Finally, we find that, by passively cross-linking long saturated tail-containing GPI-APs, the cell-spreading response may be activated even in the absence of integrin ligands, implicating clustering in regulating integrin function.

## Results

### Integrin Activation Generates Nanoclusters of the Outer-Leaflet GPI-APs in Living Cells

**Figure 1:**
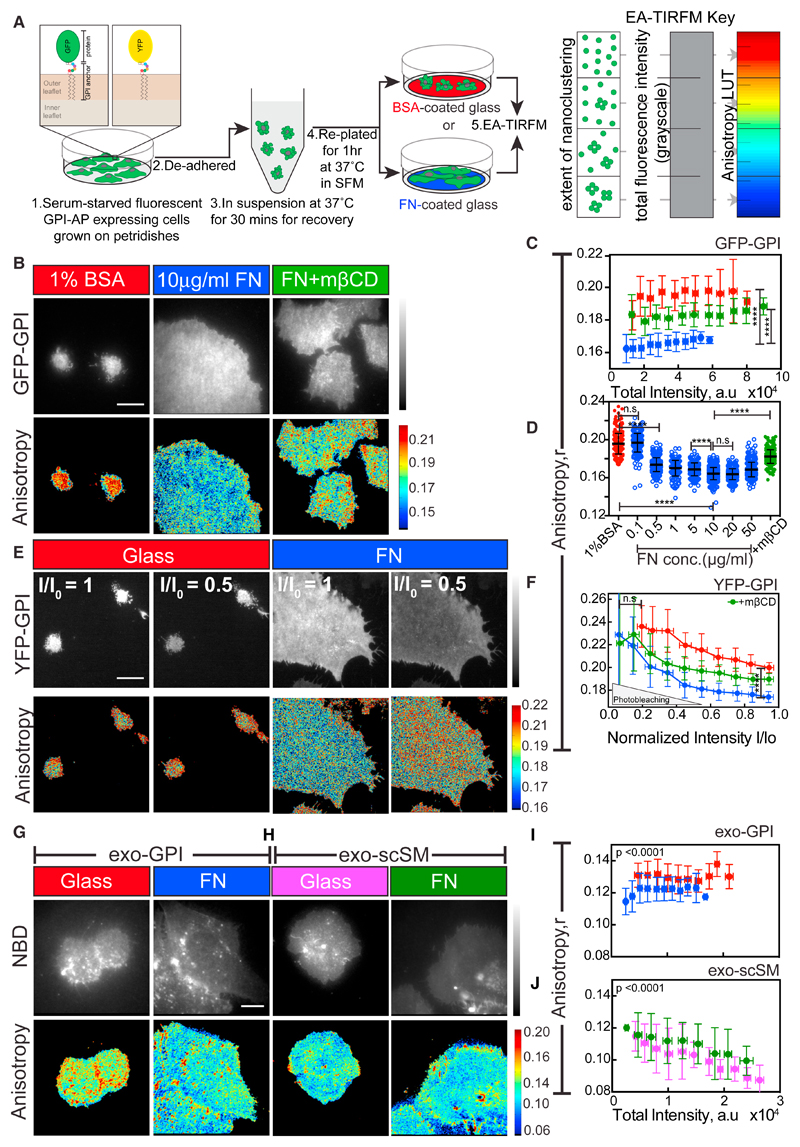
Activation of Fibronectin Binding Integrins Leads to Enhanced Nanoclustering of GPI-APs in Living Cells (A) Left: experimental schema: GPI-AP-transfected cells were de-adhered and re-plated on glass coverslips with the indicated coatings in serum-free media (SFM). The inset shows GFP or YFP-GPI at the outer leaflet of the PM. Right: in the absence of other contributing factors, the change in anisotropy value of fluorescently tagged GPI-APs reports on the extent of homoFRET due to the proximity of like fluorophores detected using an emission anisotropy TIRF microscope (EA-TIRFM). By convention, anisotropy maps are color coded, with low (or high) anisotropy denoted by blue (or red) pixels that correspond to regions enriched (or depleted) in nanoclusters. Representative intensity and steady-state anisotropy images are shown (B, E, G, and H). (B–D) GFP-GPI-expressing cells re-plated for 1 h on glass coverslips coated with 1% BSA (red) or 10 μg/mL FN before (blue) or after treatment with 10 mM mβCD for 45 min (green) (B). Graphs show plots of mean anisotropy at various total intensity bins (C) and scatter dot plots with mean anisotropy values (D) of regions of interest (ROIs) obtained from cells plated on BSA-glass (red) or indicated concentrations of FN before (blue) or after treatment with mβCD (green). Note: GPI anisotropy data are represented as scatter dot plots, when fluorescence emission anisotropy is independent of its total intensity. (E and F) YFP-GPI-expressing CHO cells plated on glass (red) or FN before (blue) (E) or after treatment with mβCD (green) and corresponding anisotropy versus relative intensity (*I/Io*) plots (F) during photobleaching. (G–J) CHO cells labeled with the GPI-analog NBD-GPI (exo-GPI: exogenous GPI; G) or C_6_NBD-sphingomyelin (exo-scSM; H) and re-plated on glass (red, magenta) or on FN (blue, green). Plots in (I) and (J) show mean anisotropy at various total intensity bins. Note that, unlike exo-GPI, the exo-scSM exhibited a concentration-dependent anisotropy with lower anisotropy on glass. Scale bar, 10 μm in all panels. All the error bars represent SD. Not significant (n.s.) p > 0.05, *p ≤ 0.05, **p ≤ 0.01, ***p ≤ 0.001, ****p ≤ 0.0001. Sample size and p values are provided in Table S4. See also Figure S1.

Integrins bind extracellular ligands, activating downstream structural and signaling molecules (Hynes, 2002; Vicente-Manzanares et al., 2009). ICAM-1 binding to its integrin receptor LFA-1 in immune cells results in hotspots of GPI-AP nanoclusters at the site of activation (van Zanten et al., 2009). To see whether activation of other integrins also leads to GPI-AP nanoclustering, we used fluorescence emission anisotropy-based microscopy to assess the extent of resonance energy transfer between like fluorophores tagged to GPI-APs (homoFRET). Nanoscale clustering increases homoFRET and decreases fluorescence emission anisotropy, allowing us to monitor nanoclustering in living cells (Ghosh et al., 2012). Chinese hamster ovary (CHO) cells stably expressing EGFP (GFP) or YFP-tagged GPI were de-adhered and re-plated on glass coated with fibronectin (FN) or BSA (Figure 1A). FN engages with a specific integrin subset that promotes cell spreading (Hynes, 2002), whereas the BSA surface does not (Figure 1B).

Although the fluorescence intensity (i.e., concentration) of GFP-GPI at the cell surface was comparable, the fluorescence emission anisotropy was much lower in spread cells on FN compared to those plated on BSA-coated glass (Figures 1B and 1C). This decrease in emission anisotropy occurred in a FN-concentration-dependent and saturable manner (Figure 1D). To ascertain the cause for the decrease in anisotropy, we photobleached YFP-GPI-expressing cells plated on FN. This resulted in a linear increase in anisotropy concomitant with a reduction in fluorescence intensity (Figures 1E and 1F). This is consistent with the existence of a high fraction of nanoclusters (Sharma et al., 2004). The higher initial value of anisotropy, and minimal change upon photo-bleaching, confirms the very low fraction of nanoclusters that form in cells plated on glass (Figures 1E, 1F, S2A, and S2B). The YFP-GPI anisotropy upon photobleaching in all conditions converged to a similar value of ~0.23, corresponding to the emission anisotropy of monomers, indicating homoFRET as the sole contributing factor to the observed decrease in anisotropy on FN.

The decrease in anisotropy when cells were plated on FN was also sensitive to the cholesterol-sequestering agent methyl-β-cyclodextrin (mβCD), that disrupts nanoscale clustering of GPI-APs (Varma and Mayor, 1998; Figures 1B–1D). This decrease in anisotropy on FN occurred for an exogenously incorporated fluorescent GPI analog (NBD-GPI) (Figures 1G and 1I; exo-GPI; Raghupathy et al., 2015), whereas an exogenously incorporated fluorescent short acyl-chain-containing sphingomyelin analog (C_6_-NBD-SM) did not exhibit the same behavior (Figures 1H and 1J; exo-scSM). This indicates that, unlike *passive* molecules like GPI-APs, *inert* molecules (short acyl chain-containing lipids) do not exhibit an increase in nanoclustering on FN.

To discern which integrin sub-types were involved in the generation of GPI-AP nanoclusters, we utilized function-perturbing antibodies directed against the αv(β3) class and the (α5)β1 class of FN-binding integrins (Byron et al., 2009; Leiss et al., 2008) or plated cells on diverse integrin ligands (Hynes, 2002). Pre-treatment with an activity-blocking β1 integrin antibody (4B4) resulted in the failure of U2OS cells to spread and generate GPI-AP nanoclusters on FN (Figures S1A and S1B). Blocking function of the αv class of integrins (17E6) (Figures S1E–S1G) or pre-treatment of cells with a non-function-perturbing β1 integrin antibody (K20) or with an antibody against an unrelated transmembrane protein such as the transferrin receptor (OKT9) did not have a similar effect (Figures S1A–S1D). Compared to effects observed on FN, U2OS cells plated on Poly-L-Lysine (that permits integrin-independent adhesion; Schlaepfer et al., 1994) or on Laminin, Collagen-1, or Vitronectin (all of which engage different integrin subtypes; Hynes, 2002) do not exhibit a significant reduction in GPI anisotropy (Figures S1H and S1I) indicating a specific response to FN-binding integrins.

We next tested whether merely activating integrins promotes nanoclustering. For this, we plated U2OS cells on low concentrations of FN in the presence of increasing amounts of Mn^2+^, a treatment that potentiates integrin activation (Mould et al., 2002). This resulted in a dose-dependent decrease in GPI-AP anisotropy, whereas, on higher concentrations of FN, addition of Mn^2+^ did not result in a further decrease (Figures S1J and S1K). Together, these data indicate that the enhanced nanoclustering on FN is mediated by the activated β1 class of integrins, and shifting the equilibrium toward a ligand-engaged integrin, either by increasing FN density or by activation through Mn^2+^, promotes the generation of GPI-AP nanoclusters.

### Generation of GPI-AP Nanoclusters Precedes the Rapid Cell-Spreading Phase

**Figure 2:**
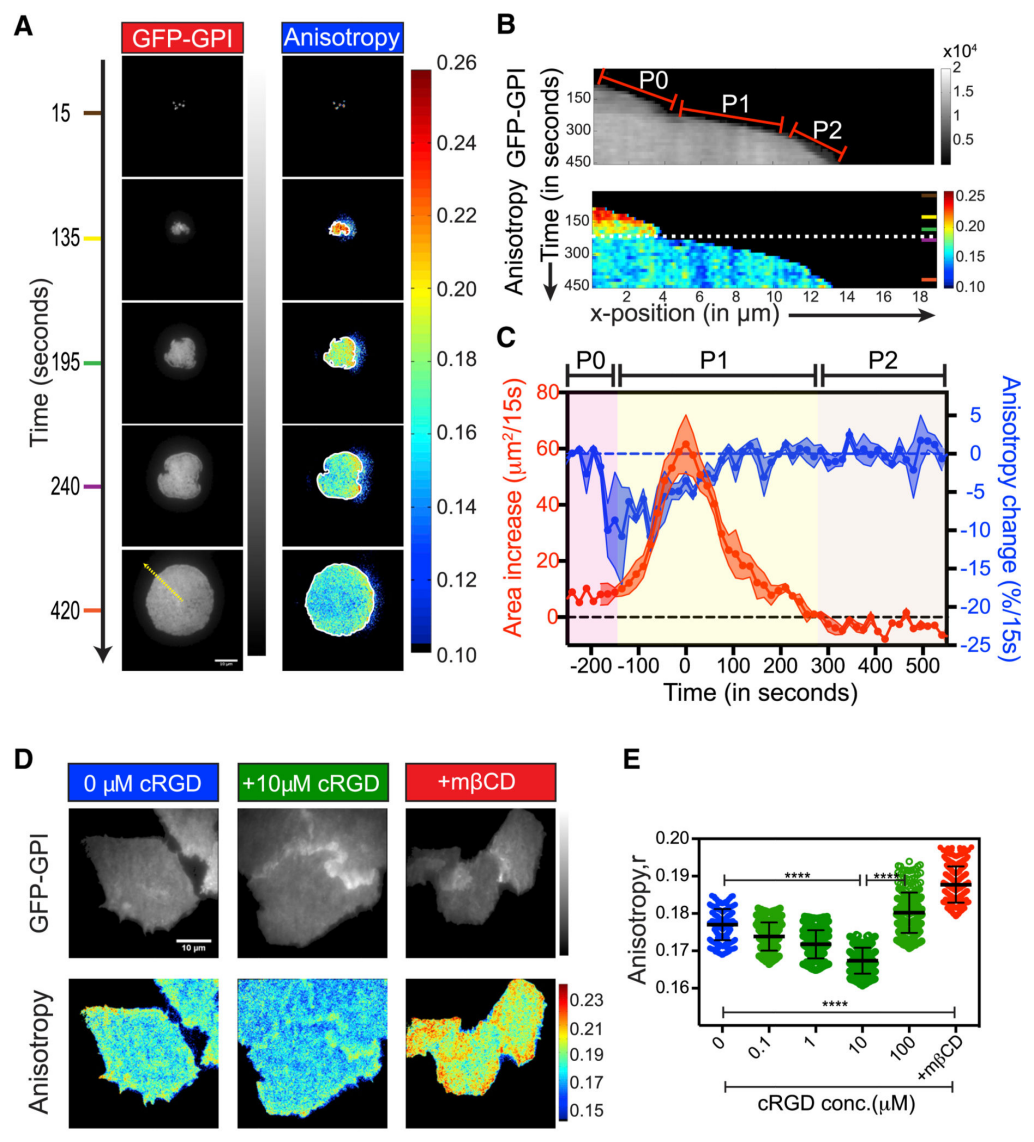
Activation of RGD Binding Integrins Leads to Enhanced Nanoclustering of GPI-APs during the Early Stages of Cell Spreading (A) Representative intensity and steady-state anisotropy images of GFP-GPI-expressing CHO cells at the indicated times post-settling on FN. (B) Kymograph shows the time trace of GPI intensity (top) and anisotropy (bottom) of a 3 pixel line (ROI) drawn perpendicular to the cell edge (yellow line in A) exhibiting cell-spreading phases (red lines in top panel); P0–P1 transition (dashed white line in bottom panel) is shown. (C) Graph shows the change in the whole cell area (red curve; left y axis) and the corresponding normalized GFP-GPI anisotropy change (blue curve; right y axis) between two consecutive frames 15 s apart, plotted as a function of spreading time. The solid white lines in (A) denote the segmented outline of cells used in the graph. Data from 4 cells have been aligned relative to the timing of the peak area change (t = 0 in C). Dots represent the mean for each time bin; shaded region marks the SEM. (D and E) GFP-GPI-expressing CHO cells grown on glass coverslips and imaged directly (blue) or after incubation (30 min) with indicated concentrations of soluble cRGD (green) either before or after treatment with 10 mM mβCD (red). Scatter dot plot (E) with mean anisotropy values. Note that at high concentrations of cRGD (>100 μM) cell detach due to ligand competition, resulting in higher anisotropy values. Scale bar, 10 μm in all panels. Error bars represent SD. n.s. p > 0.05, *p ≤ 0.05, **p ≤ 0.01, ***p ≤ 0.001, ****p ≤ 0.0001. Sample size and p values are provided in Table S4. See also Figure S2 and Video S1.

When plated on FN, cells undergo three major phases of spreading behavior; Phase 0 (P0), the wetting phase mediated by the initial engagement of the integrin with its ligand; Phase 1 (P1), the rapid expansion phase where cells spread vigorously to establish a large contact area; and finally Phase 2 (P2), where myosin II contractility based probing of the substrate via periodic protrusion and retraction of the cell edge senses substrate rigidity and asymptotic spreading continues until maximum area is attained (Figure 2B, top panel). These phases define critical checkpoints for progression from a suspended state to a fully spread state on FN (Wolfenson et al., 2014) and provided us with a tractable assay system to correlate the universal characteristics of cell spreading with changes in GPI-AP nanoclustering.

As the cell first comes in contact with the FN, it appeared to be devoid of nanoclusters (Figures 2A and 2B; Video S1) and rapidly began to acquire nanoclusters (blue pixels) co-incident with the P0-P1 transition phase (Figures 2B, 2C, and S2C). There was a sudden decrease in the anisotropy of GFP-GPI (Figures 2C and S2D) that preceded the peak in cell expansion characteristic of the P2 phase by ~150 s (Figure 2C). This decrease in GFP-GPI anisotropy persisted for ~300 s, beyond which no further decrease occurred, although the increase in cell area continued for up to ~450 s (Figure 2C).

The time from the initial contact until initiation of cell spreading was shown to be inversely correlated with the FN density (Dubin-Thaler et al., 2004), suggesting that this process is triggered by the integration of chemical signals via integrin activation. To test whether GPI-AP nanoclustering is also an integral response of such a chemical signaling process, we treated adhered cells with soluble cyclic Arg-Gly-Asp peptide (cRGD). RGD is the sequence motif on FN recognized by integrins that activates several downstream signaling molecules (Ruoslahti, 1996; Zhang et al., 2014). We observed a dose-dependent decrease in GPI-AP anisotropy either in cells plated on glass for 2 days and treated with the soluble cRGD peptide (Figures 2D and 2E) or in cells that were freshly plated onto cRGD immobilized onto glass surfaces (Figures S2E–S2G) indicating that the increase in GPI-AP nanoclustering was indeed triggered by integration of a chemical signaling response initiated by integrin-ligand binding.

### An SFK-FAK-RhoA Signaling Cascade Generates GPI-AP Nanoclusters

**Figure 3:**
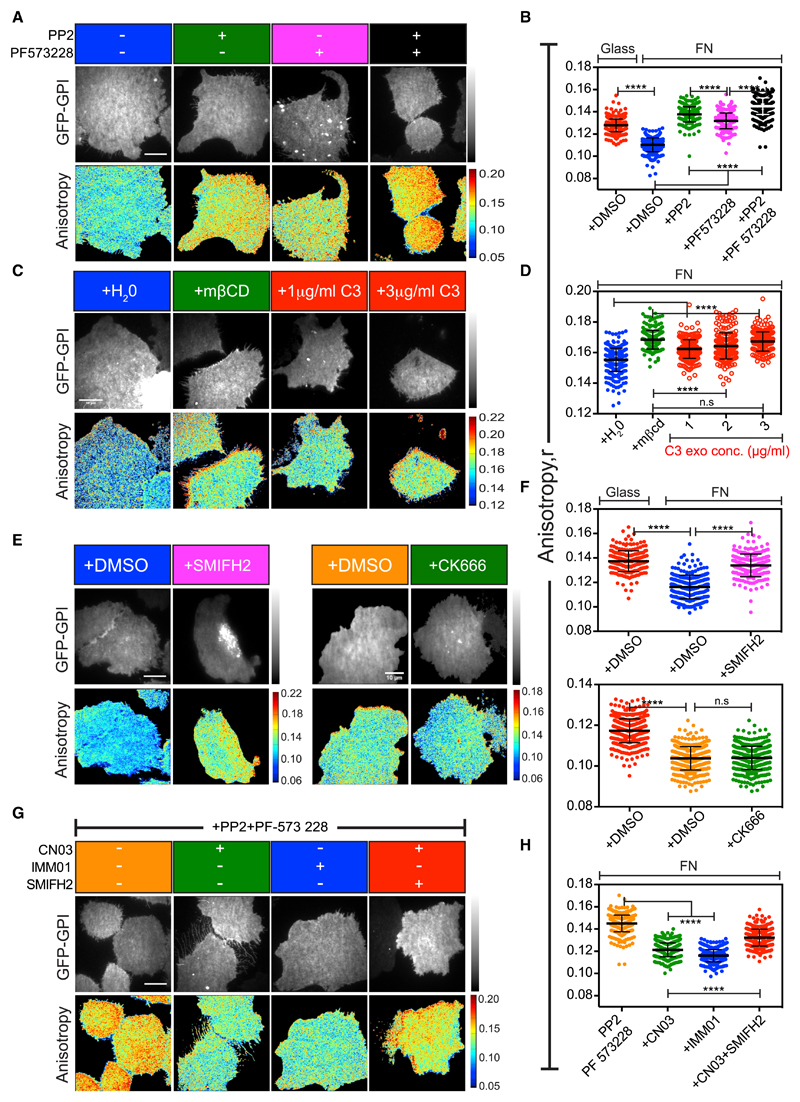
Inhibition of SFK-FAK, RhoGTPase, and Formins Leads to Loss of FN-Triggered Nanoclustering of GPI-APs (A–H) Representative intensity and steady-state anisotropy images and scatter dot plots with mean anisotropy values of ROIs obtained from GFP-GPI-expressing CHO cells re-plated on FN and imaged following pre-treatment with the following: (A and B) DMSO (blue), SFK inhibitor PP2 (20 μM; green), FAK inhibitor PF-573 228 (10 μM; magenta) or both (black); (C and D) indicated concentrations of RhoA inhibitor exoenzyme C3 transferase (C3 exo; red) or with the vehicle (H_2_O;blue) or with mβCD (10 mM, green); (E and F) formin inhibitor SMIFH2 (10 μM, magenta; top in F) or Arp2/3 inhibitors CK666 (100 μM, green, bottom in F) or respective DMSO vehicle control (blue, orange); (G and H) formin agonist CN03 (1 μg/mL, green) or RhoA activator IMM01 (10 μM, blue) or with RhoA activator and SMIFH2 (red), all in the presence of 20 μM PP2 and 10 μM PF-573 228. DMSO pre-treated cells on glass is shown in red in (B) and (F). Scale bar, 10 μm in all panels. All error bars represent SD. n.s. p > 0.05, *p ≤ 0.05, **p ≤ 0.01, ***p ≤ 0.001, ****p ≤ 0.0001. Sample size and p values are provided in Table S4. See also Figure S3.

GTPases, tyrosine kinases, and various bona fide cytoskeletal modifying proteins are involved in integrin-mediated responses (Vicente-Manzanares et al., 2009). To investigate the signaling pathway that leads to GPI-AP nanoclustering, we employed a chemical and genetic perturbation approach. Treatment of cells with the SFK inhibitor PP2 (Hanke et al., 1996) and the FAK inhibitor PF 573 228 (Slack-Davis et al., 2007) resulted in loss of GPI-AP nanoclustering on FN (Figures 3A and 3B), implicating these integrin signaling activated kinases in the process. Correspondingly, FAK-deficient fibroblasts (llić et al., 1995) also failed to support GPI-AP nanoclustering on FN (Figures S3A and S3B).

A downstream target of the SFK and FAK kinases is the small GTPase RhoA (Guilluy et al., 2011). Increasing concentrations of the cell-permeable RhoA inhibitor C3 exoenzyme (Aktories et al., 1987; Braun et al., 1989) inhibited GPI-AP nanoclustering on FN in a dose-dependent manner (Figures 3C and 3D). Reciprocally, addition of a cell-permeable RhoA activator (CN03; Flatau et al., 1997; Schmidt et al., 1997) induced enhanced nanoclustering of GPI-APs even when cells were plated on plain glass, a condition where there was minimal integrin activation and corresponding changes in cell-spread area (Figures S3C–S3E). Furthermore, the failure of FN engagement in promoting nanoclustering when cells were treated with both SFK-FAK inhibitors was rescued by the ectopic activation of RhoA (Figures 3G and 3H). These results demonstrate that RhoA operates downstream of SFK-FAK in the pathway that mediates nanoclustering of GPI-APs.

### Formin-Based Actin Nucleators Are Necessary for GPI-AP Nanoclustering

We next investigated the role of the actin-nucleators that are downstream targets of integrin activation in mediating the nanoclustering of GPI-APs. Treatment of cells with the formin inhibitor SMIFH2 (Rizvi et al., 2009) but not the Arp2/3 complex inhibitor CK666 (Nolen et al., 2009) resulted in a loss of nanoclustering of GPI-APs on FN (Figures 3E and 3F). Moreover, the acute loss of GPI-AP nanoclusters observed after cells were treated with SFK-FAK inhibitors was reversed by treatment with a formin agonist (IMM01; Lash et al., 2013; Figures 3G and 3H). This rescue in nanoclustering could be blocked by SMIFH2 treatment (Figures 3G and 3H), suggesting that formins were indeed the downstream targets of SFK-FAK in the pathway that mediated nanoclustering of GPI-APs. In addition, treatment of cells plated on glass with the formin agonist resulted in an increase in nanoclustering of GPI-APs even in the absence of integrin ligand engagement and correlative changes in spread area (Figures S3F–S3H). Taken together, these results indicate that actin filaments nucleated by formin are involved in the FN-induced nanoclustering of GPI-APs.

To investigate the identity of the formin that mediates the nanoclustering of GPI-APs on FN, we employed an RNAi-based approach to deplete two candidate formins mDia1 (DIAPH1) and FHOD1 that had been previously implicated in SFK and RhoA/ROCK-dependent cell motility (Koka et al., 2005; Takeya et al., 2008; Watanabe et al., 1997). Although we observed a decrease in nanoclustering of GPI-APs with RNAi directed against both the formin members, the defect was more prominent in the case of FHOD1 depletion (Figures S4C and S4D), despite there being ~60% compensatory increase in levels of mDia1under this condition (Figures S4A and S4B). This implicates FHOD1 as one of the major formin members involved in this process.

### Integrin Activation Triggers an Acto-myosin-Based Clustering Mechanism

**Figure 4:**
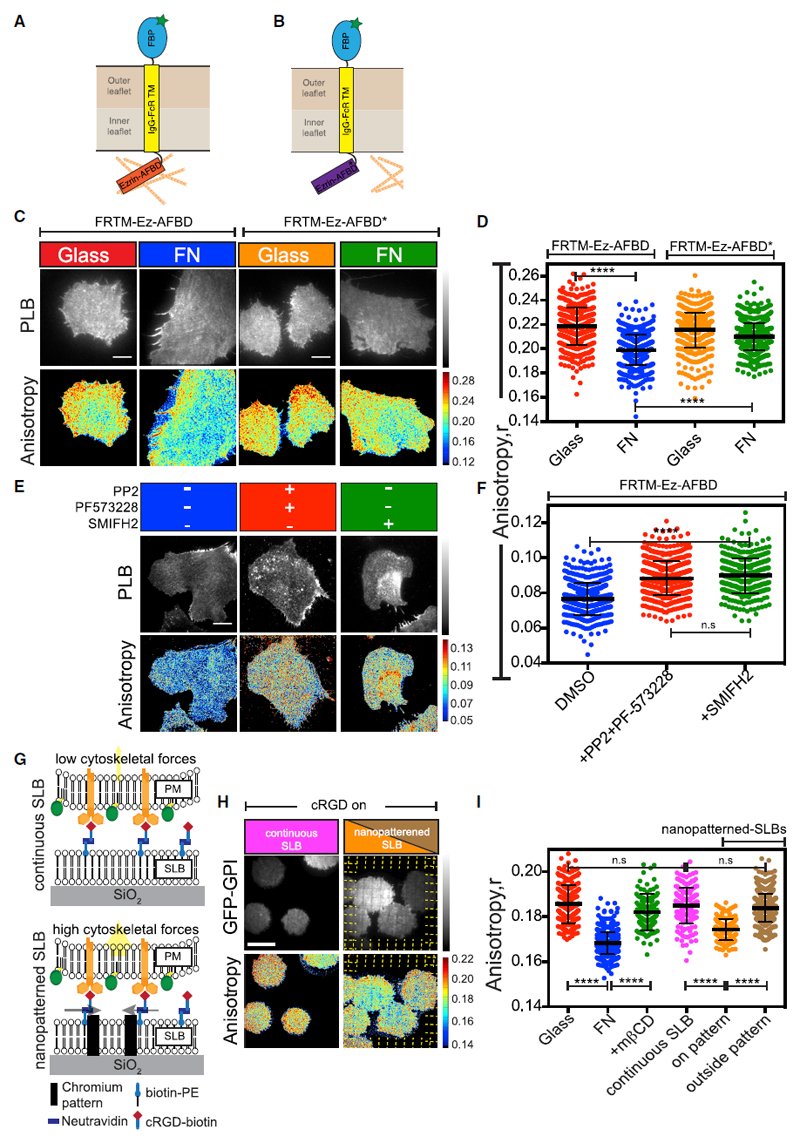
Integrin Activation Triggers Changes in Dynamic Actin Activity and Requires cRGD Ligand Immobilization (A and B) Schematic of the model transmembrane protein FRTM-Ezrin-AFBD (A) and the mutant FRTM-Ezrin-R579A (FRTM-Ez-AFBD*) (B) that impairs Ezrin-AFBD ability to interact with actin (Gowrishankar et al., 2012). (C–F) Representative intensity and steady-state anisotropy images and scatter dot plots with mean anisotropy of ROIs obtained from CHO cells stably expressing either FRTM-Ez-AFBD or FRTM-Ez-AFBD* as indicated. The cells were labeled with fluorescent folate, Pteroyl-lysyl-Bodipy(PLB) and plated on FN (blue, green) or glass (red, orange) prior to imaging in the absence (C and D) or after pre-treatment (E and F) with either 20 μM PP2 and 10 μM PF-573228 (red) or 10 μM SMIFH2 (green) or with the vehicle (DMSO; blue). (G–I) Schematic (G) of the supported lipid bilayer functionalized with cRGD that was prepared either on plain (continuous SLB; top) or on 5-nm-tall and 100-nm-wide chromium patterned (nanopatterned SLB, bottom) glass surfaces. (H and I) GFP-GPI-expressing CHO cells plated on glass (red) or on FN (blue) or treated with 10 mM mβCD on FN (green) or plated on either continuous SLBs with mobile ligand (magenta) or SLBs assembled on chromium nano-patterned surfaces. ROIs were drawn either on the pattern (orange) where the ligand is transiently immobile or from regions outside (brown) where the ligand is mobile. Scale bar 10μm in all panels. All error bars represent SD. n.s. p > 0.05, *p ≤ 0.05, **p ≤ 0.01, ***p ≤ 0.001, ****p ≤ 0.0001. Sample size and p values are provided in Table S4. See also Figure S4.

To test whether integrin signaling resulted in the activation of a cortical acto-myosin based nanoclustering machinery that had been previously described (Gowrishankar et al., 2012), we monitored the organization of the actin filament binding domain (AFBD)-containing transmembrane receptor FRTM-Ez-AFBD (Figure 4A). The nanoclustering of this chimeric receptor solely depends on its ability to associate with the dynamic acto-myosin machinery (Gowrishankar et al., 2012) and thus serve as reporters of this activity. Similar to GPI-APs, FRTM-Ez-AFBD showed a decrease in its anisotropy when plated on FN (Figures 4C and 4D). By contrast, cells expressing FR-TM with a mutated version of the Ez-AFBD (R579A) that has reduced binding to actin (FRTM-Ez-AFBD*; Figure 4B; Gowrishankar et al., 2012) did not display a similar extent of change when plated on FN (Figures 4C and 4D).

The detection of a pool of slowly diffusing actin filaments bound to the AFBD derived from the cytoskeletal protein utrophin (UtrAFBD; Gowrishankar et al., 2012), by fluorescence correlation spectroscopy (FCS)-based measurements, provides a signature of the dynamic actin machinery that drives nanoclustering of membrane proteins (Saha et al., 2015). When the diffusion of GFP-UtrAFBD was monitored by FCS traces taken from cells plated on FN, in regions at the periphery that were devoid of stress fibers (circular regions in Figure S4E), we detected at least two diffusing species (Figure S4F), one corresponding to the diffusion timescale of unbound GFP-UtrAFBD (0.3 ms < τ < 3 ms) and another slower component (τ > 10 ms) that corresponds to those bound to actin filaments with an approximate filament length of ~200 nm (Gowrishankar et al., 2012). Treatment of these cells with the formin inhibitor SMIFH2 resulted in a loss of the slow diffusing component (Figure S4F). Additionally, the nanoclustering of the chimeric transmembrane receptor was also abrogated upon inhibition of SFK-FAK as well as formins that generate the dynamic actin machinery (Figures 4E and 4F).

We next tested the role of integrin-induced myosin activation in GPI-AP nanoclustering. The Rho kinase ROCK stimulates myosin light chain (MLC) phosphorylation either directly (Amano et al., 1996) or indirectly via the inhibition of MLC phosphatase (Kimura et al., 1996). Inhibition of ROCK using the Y-27632 inhibitor (Uehata et al., 1997) or MLC phosphorylation with the MLC kinase (MLCK) inhibitor, ML-7 (Saitoh et al., 1987), resulted in the loss of GPI-AP nanoclusters on FN (Figures S4G and S4H).

Together, these data provide evidence that integrin signaling mediated by SFK-FAK-RhoA axis couples integrin ligation to the generation of dynamic acto-myosin machinery, which in turn promotes the nanoclustering of GPI-anchored and other membrane proteins that can engage with this active machinery.

### GPI-AP Nanoclustering Requires an Immobilized Integrin Ligand

Integrin engagement and clustering are early steps in the formation of cell-substrate adhesions and occur independent of force (Choi et al., 2008). Cells plated on supported lipid bilayers (SLBs) functionalized with RGD ligand that was mobile within the bilayer plane (continuous SLB; Figure 4G top) formed sub-micron-sized α5(β1) integrin clusters that co-localized with RGD complexes on the SLBs (Figure S4I). These clusters recruited FA adaptor proteins such as talin, src, FAK, and nucleate FHOD1-generated actin filaments in a force-independent manner (Changede et al., 2015; Iskratsch et al., 2013; Yu et al., 2011). Since several of these molecules were also implicated in the pathway that generates the nanoclusters of GPI-APs, we tested whether integrins bound to mobile ligands in the absence of force on such continuous SLBs were sufficient to drive GPI-AP nanoclustering. Surprisingly, the extent of nanoclustering observed when cells were plated on these surfaces were significantly different from those plated on FN and resembled cells plated on uncoated glass (Figures 4H and 4I), indicating minimal activation of the nanoclustering mechanism when the integrin ligand is mobile.

In some instances, however, the cells exhibited lower anisotropy (data not shown). We reasoned that this resulted from imperfections on the SLB that serve to immobilize the ligand (see STAR Methods and Yu et al., 2011). To test whether GPI nanoclustering required ligand immobilization, we plated cells on SLBs that were prepared on chromium patterned (5 nm tall and 100 nm wide) substrates, where the lines deliberately offered resistance to the long-range lateral mobility of the ligand (nanopatterned SLB; schematic in Figure 4G, bottom; STAR Methods; Yu et al., 2011). Strikingly, we observed a decrease in GFP-GPI anisotropy in a patchy manner and extending ~0.5 μm on either side of the patterns (Figures 4H and 4I). The decrease in anisotropy was not observed in the inter-pattern regions where the SLB was continuous and the ligand was mobile (Figures 4H and 4I), was independent of the total intensity of GFP-GPI (Figure S4J), and was not observed when anisotropy of a thin film of purified GFP solution was measured on or away from the pattern (Figures S4K and S4L).

Taken together, this suggests that ligand immobilization, which results in a force-dependent maturation of the integrin clusters adjacent to the patterns (Yu et al., 2011), leads to a robust and localized activation of the GPI-AP nanoclustering mechanism.

### Talin and Vinculin Are Necessary for the Generation of GPI-AP Nanoclusters

The force-dependent activation of integrin signaling is accompanied by the recruitment of talin and vinculin, components of the mechano-sensing system of integrin-based FAs (Atherton et al., 2016). We thus tested the role of talin and vinculin in the generation of GPI-AP nanoclusters on FN.

**Figure 5:**
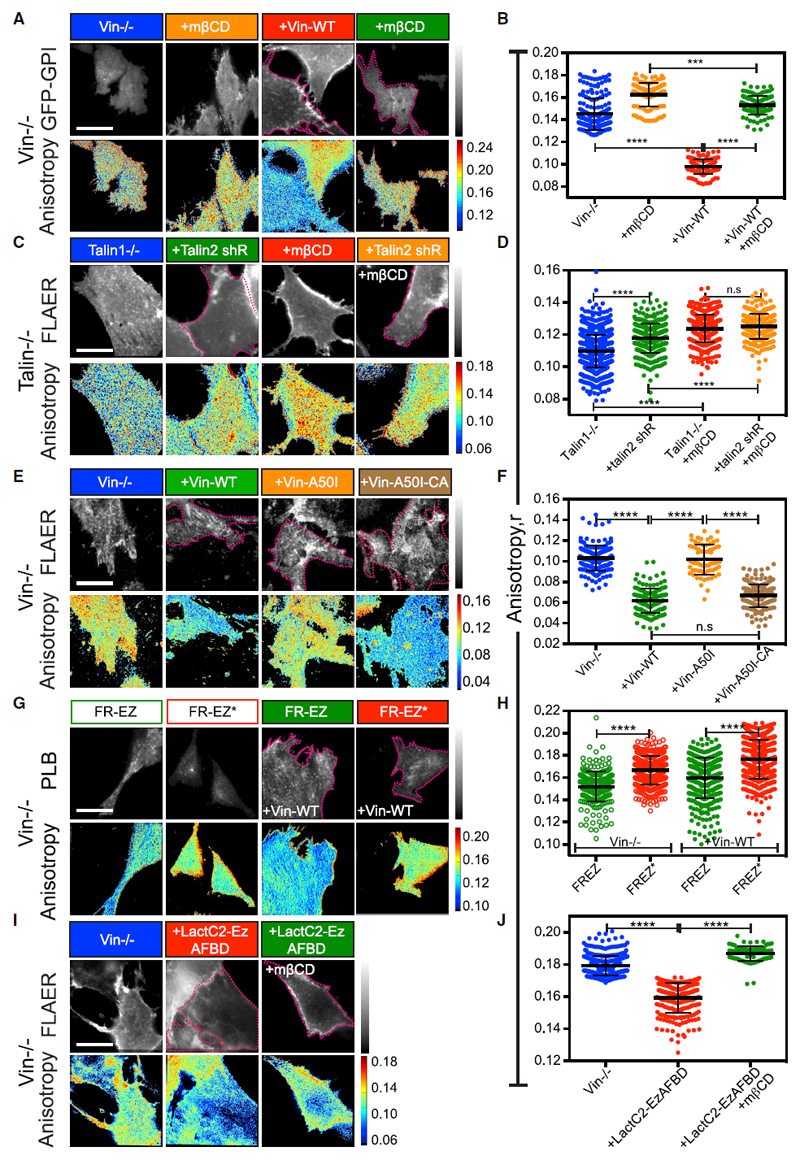
Talin and Vinculin Are Required for Facilitating GPI-AP Nanoclustering in Mouse Embryonic Fibroblasts (A–J) Representative intensity and steady-state anisotropy images (A, C, E, G, and I) and scatter dot plot with mean anisotropy values (B, D, F, H, and J) of ROIs obtained from (A and B) vinculin-deficient cells (Vin^−/−^) transfected with GFP-GPI (blue, orange) or co-transfected with mCherry-vinculin (+Vin-WT; red, green) and plated on FN or subsequently treated with 10 mM mβCD (orange, green). (C and D) Talin1-deficient cells without (Talin1^−/−^; blue, red) or with co-transfection with Talin2 shRNA (+Talin2 shR; green, orange) and re-plated onto FN after labeling with Alexa-568-FLAER prior to (blue, green) or post-treatment with 10 mM mβCD (red, orange). (E and F) Vin^−/−^ cells alone (blue) or transiently transfected with GFP tagged Vin-WT (green), Vin-A50I (orange), or Vin-A50I-CA (brown) and plated onto FN after labeling with Alexa-568-FLAER. (G and H) Vin^−/−^ cells were transiently transfected with FRTM-Ez-AFBD (FR-EZ; green) or with FR-Ez-AFBD* mutant (FR-EZ*; red), without (open circles) or with Vin-WT (closed circles) and re-plated onto FN after labeling with PLB. (I and J) Vin^−/−^ cells alone (blue) or transfected with Lact C2-Ez-AFBD YFP (red) were labeled with Alexa-568-FLAER and re-plated on FN and directly labeled or treated with 10 mM mβCD (+mβCD; green). Dotted magenta lines in all images outline the transfected cells expressing the indicated constructs. Scale bar, 10 μm in all panels. All error bars represent SD. n.s. p > 0.05, *p ≤ 0.05, **p ≤ 0.01, ***p ≤ 0.001, ****p ≤ 0.0001. Sample size and p values are provided in Table S4. See also Figure S5.

Vinculin knockout (Vin^−/−^) mouse embryonic fibroblasts (MEFs) transfected with GFP-GPI and freshly plated on FN exhibited a high anisotropy value, which was largely unaffected by mβCD treatment (Figures 5A and 5B), indicating minimal GPI-AP nanoclustering under this condition. To restore vinculin function, we transfected full-length vinculin into Vin^−/−^ MEFs. This resulted in increased GPI-AP nanoclustering that was additionally sensitive to cholesterol removal by mβCD (Figures 5A and 5B). These data indicate that the absence of vinculin disrupts GPI-AP nanoclustering on FN.

Vinculin activation requires talin (Case et al., 2015). Therefore, we tested the role of talin in GPI-AP nanoclustering in talin-deficient cells (Talin^−/−^). Since loss of talin1 leads to overexpression of the talin2 isoform (Zhang et al., 2008), we additionally depleted talin2 with a talin2 short hairpin RNA (shRNA) co-expressed with GFP in these cells (Figures S5A and S5B). The GPI anisotropy was indeed higher in talin2 shRNA-expressing Talin1^−/−^ cells when compared to the Talin-1^−/−^ cells (Figures 5C and 5D), consistent with the requirement of talin in the generation of GPI-AP nanoclusters. However, this increase was less than that observed for the loss of vinculin; the partial loss of talin2 (Figures S5A and S5B) could serve as a confounding factor in these experiments.

To test whether vinculin operates downstream of talin, we expressed Vin-A50I, a mutant incapable of binding talin (Figure S5C). This mutant failed to support GPI-AP nanoclustering when expressed in Vin^−/−^ cells (Figures 5E and 5F), suggesting that talin binding capacity is necessary for vinculin to restore GPI-AP nanoclustering in these cells. Strikingly, a constitutively activated version of the same, Vin-A50I-CA (Figure S5C), which no longer requires talin for its activation, restored GPI-AP nanoclustering in the Vin^−/−^ MEFs (Figures 5E and 5F). Together these results show that GPI-AP nanoclustering on FN normally requires talin-mediated vinculin activation.

### Vinculin Is Required for Coupling GPI-APs to the Actomyosin-Based Clustering Machinery

We next asked whether vinculin was involved in triggering the actomyosin-based clustering machinery downstream of integrin activation. For this purpose, we examined the nanoclustering status of the FRTM-Ez-AFBD construct in Vin^−/−^ cells. Surprisingly, FRTM-Ez-AFBD exhibited a lower anisotropy value compared to the FRTM-Ez-AFBD* in Vin^−/−^ cells as well as in the full-length vinculin-restored cells (Figures 5G and 5H), indicating that the Vin^−/−^ cells were not defective in generating the acto-myosin machinery. Furthermore, vinculin re-expression-mediated restoration of GPI-AP nanoclustering in Vin^−/−^ MEFs was completely disrupted upon pre-treatment with the SFK and FAK inhibitor PP2 and PF 573228, respectively, or with the formin inhibitor SMIFH2 (Figures S6A and S6B). GPI-AP nanoclustering was only marginally restored by treatment of Vin^−/−^ cells with the formin activator IMM01 (Figures S6C and S6D).

The addition of an artificial linker (LactC2-Ez-AFBD) (Raghupathy et al., 2015) that can directly link inner-leaflet PS lipids (via LactC2 domain of lactadherin) to actin (via Ezrin-AFBD) was able to restore GPI-AP nanoclustering in a cholesterol sensitive manner in Vin^−/−^ cells (Figures 5I and 5J). The extent of GPI-AP nanoclustering was similar to that observed with the re-introduction of vinculin in these cells (Figures S5D and S5E) and was dependent on its ability to bind PS; a mutant LactC2 that has reduced binding to PS but can still bind actin (LactC2-AAA-Ez AFBD; Yeung et al., 2008) failed to restore GPI-AP nanoclustering in Vin^−/−^ cells (Figures S5D and S5E).

These observations indicate that, while vinculin activation is not necessary for triggering the acto-myosin clustering machinery, it is involved in the pathway that links actin activity to the immobilization of inner-leaflet lipids.

### Lipid and Actin Binding Capacity of Vinculin Are Necessary for GPI-AP Nanoclustering

**Figure 6:**
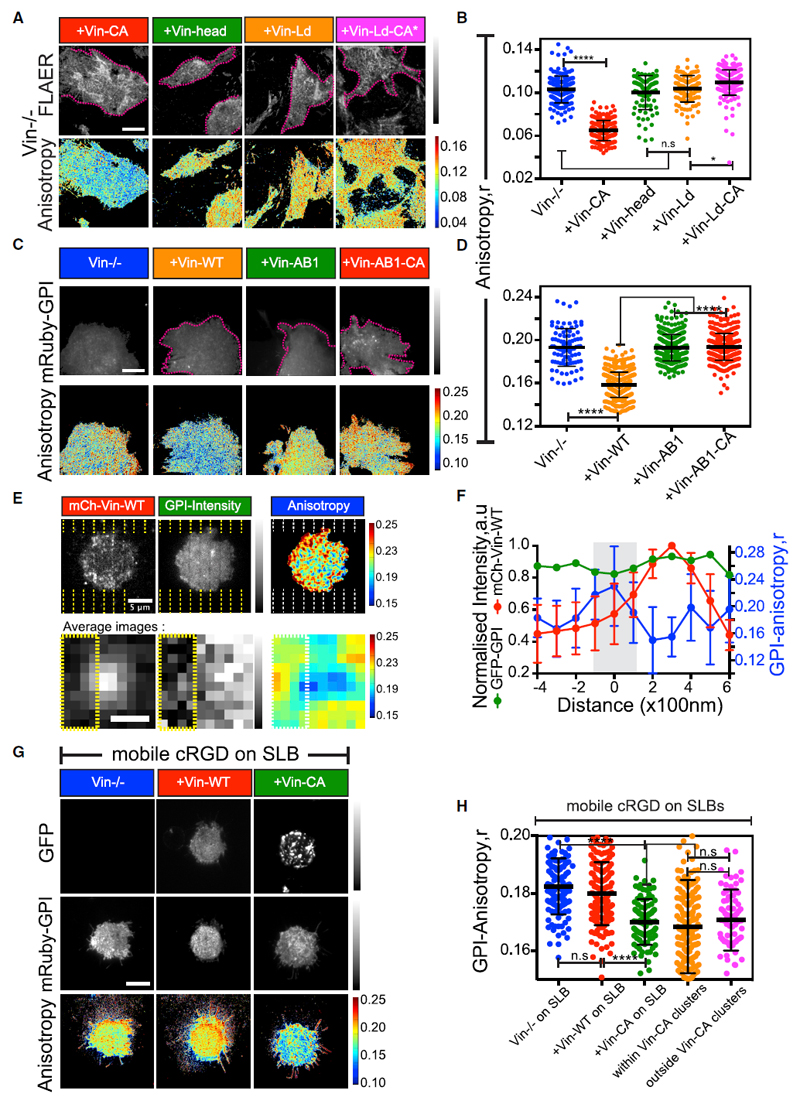
Vinculin Facilitates GPI-AP Nanoclustering in an Integrin Signaling-Dependent Manner (A–D) Representative intensity and steady-state anisotropy images and scatter dot plot with mean anisotropy values of ROIs obtained from Alexa-568-FLAER-labeled (A and B) or mRuby2-GPI (C and D)-expressing Vin^−/−^ cells or Vin^−/−^ cells transfected with GFP variants of the indicated vinculin constructs and re-plated on FN prior to imaging. Note: Vin^−/−^, blue, shows data from Figure 5F, and dotted magenta lines in (A) and (C) outline the transfected cells. (E) CHO cells transfected with mCherry-vinculin (mCh-Vin-WT) and plated on cRGD functionalized SLBs assembled on nanopatterned surfaces. The dashed line represents the location of the chromium line patterns. Bottom panel: average images of vinculin clusters and correlated GPI-intensity normalized to the maximum, alongside corresponding 3-pixel averaged GPI-anisotropy images obtained from 9 independent vinculin clusters. (F) Line profiles of normalized mCh-Vin-WT mean intensity (red curve; left-y axis in F), normalized mean GFP-GPI-intensity (green curve; left y axis in F), and mean GFP-GPI-anisotropy (blue curve; right y axis in F) obtained from 10 independent line scans drawn perpendicular to the chromium patterns and passing through the Vin-WT cluster-center. Note, the gray-shaded area (in F) and left side of yellow dotted line (in E, bottom) mark the position of the chromium pattern where the dip in GFP-GPI-intensity is observed. (G and H) mRuby2-GPI expressed in Vin^−/−^ cells alone (blue) or co-transfected with GFP-Vin-WT (red) or GFP-Vin-CA (green) (G) and plated on mobile cRGD functionalized continuous SLBs and quantified from regions obtained within segmented Vin-CA clusters (orange in H) or for ROIs drawn outside such clusters (magenta in H). Note the diffuse versus clustered distribution of Vin-WT and Vin-CA, respectively, indicating insufficient activation of Vin-WT on continuous SLBs. Scale bar, 10 μm in (A), (C), and (G) and 5 μm (E, top) and 500 nm (E, bottom). All error bars represent SD. n.s. p > 0.05, *p ≤ 0.05, **p ≤ 0.01, ***p ≤ 0.001, ****p ≤ 0.0001. Sample size and p values are provided in Table S4. See also Figure S6.

Vinculin possesses lipid and actin binding sites in its tail domain (Humphries et al., 2007). Re-introduction of a truncated version of vinculin that consisted of only the head domain (Figure S5C) failed to restore GPI-AP nanoclustering in Vin^−/−^ cells (Figures 6A and 6B). Therefore, we systematically investigated the role of lipid and actin-binding capacity of vinculin in generating GPI-AP nanoclusters. Expression of a Vin-Ld mutant that lacks the capacity to bind negatively charged phospholipids (Chandrasekar et al., 2005; Figure S5C) failed to restore GPI-AP nanoclustering in Vin^−/−^ cells (Figures 6A and 6B). To determine whether this defect resulted from the inability of Vin-Ld to get activated in the first place (Thompson et al., 2017), we generated Vin-Ld-CA* that was still unable to bind negatively charged lipids but was constitutively active (Figure S5C). This mutant also failed to restore GPI-AP nanoclustering in Vin^−/−^ MEFs (Figures 6A and 6B). To assess whether the actin-binding capacity of vinculin was also necessary for GPI-AP nanoclustering, we expressed a mutant version of vinculin, Vin-AB1, which had reduced capacity to bind to actin but localized to FAs (Case et al., 2015; Figure S5C) and Vin-AB1-CA, a constitutively active version of the same (Figure S5C). These mutant constructs of vinculin were unable to restore nanoclustering of GPI-APs in Vin^−/−^ cells (Figures 6C and 6D).

Altogether, these results indicate that both the lipid binding and actin binding capacity of vinculin are necessary to catalyze GPI-AP nanocluster formation and accounts for the key mechanistic difference between the nanoclustering of GPI-APs and that of membrane proteins with direct actin binding motifs.

### Vinculin Activation Is Required for Generating GPI-AP Nanoclusters in a Force-Dependent Pathway Downstream of Ligand Immobilization

We next tested whether vinculin activity was responsible for localized GPI-AP nanoclustering effect that was observed on the RGD functionalized SLBs prepared on the chromium nanopatterns. For this, we transfected CHO cells with mCherry-vinculin and spatially correlated the vinculin-enriched contractile microclusters that formed on the cRGD functionalized nanopatterned SLB (Figure S6G), to the GFP-GPI anisotropy. Strikingly, the sites of vinculin enrichment adjacent to patterns were indeed correlated with regions in the membrane that have more GPI-AP nanoclusters, as compared to corresponding regions outside vinculin microclusters and where the RGD ligand was mobile (Figures 6E, 6F, and S6G–S6I). Thus, the observed differences in the extent of GPI-AP nanoclustering between mobile and immobile RGD ligands could be attributed to differences in the force-dependent recruitment of mechano-sensitive components such as vinculin.

To test whether the activation of vinculin was sufficient to support GPI-AP nanocluster formation even when the ligand was mobile, we transfected Vin^−/−^ cells with either vinculin wild type (Vin-WT; Figure S5C) or vinculin constitutively active (Vin-CA; Figure S5C) mutant construct and plated them on RGD functionalized continuous SLBs. In striking contrast to observations on FN (Figure S5E), Vin-CA transfection-restored GPI-AP nanoclustering on this surface, whereas transfected Vin-WT behaved identically to the Vin^−/−^ cells (Figures 6G and 6H). In Vin-CA-transfected cells, GPI-AP nanoclustering occurred throughout the cell membrane of these cells (Figure 6H), consistent with the possibility that localized force-dependent activation of vinculin, normally restricted to immobilized integrin microclusters, was bypassed by expression of Vin-CA.

Thus, GPI-AP nanoclustering is spatially regulated and localized to sites of ligand immobilization through the force-dependent activation of vinculin.

### Cells Defective in the GPI-AP Nanocluster Formation Exhibit Aberrant Integrin Function

**Figure 7:**
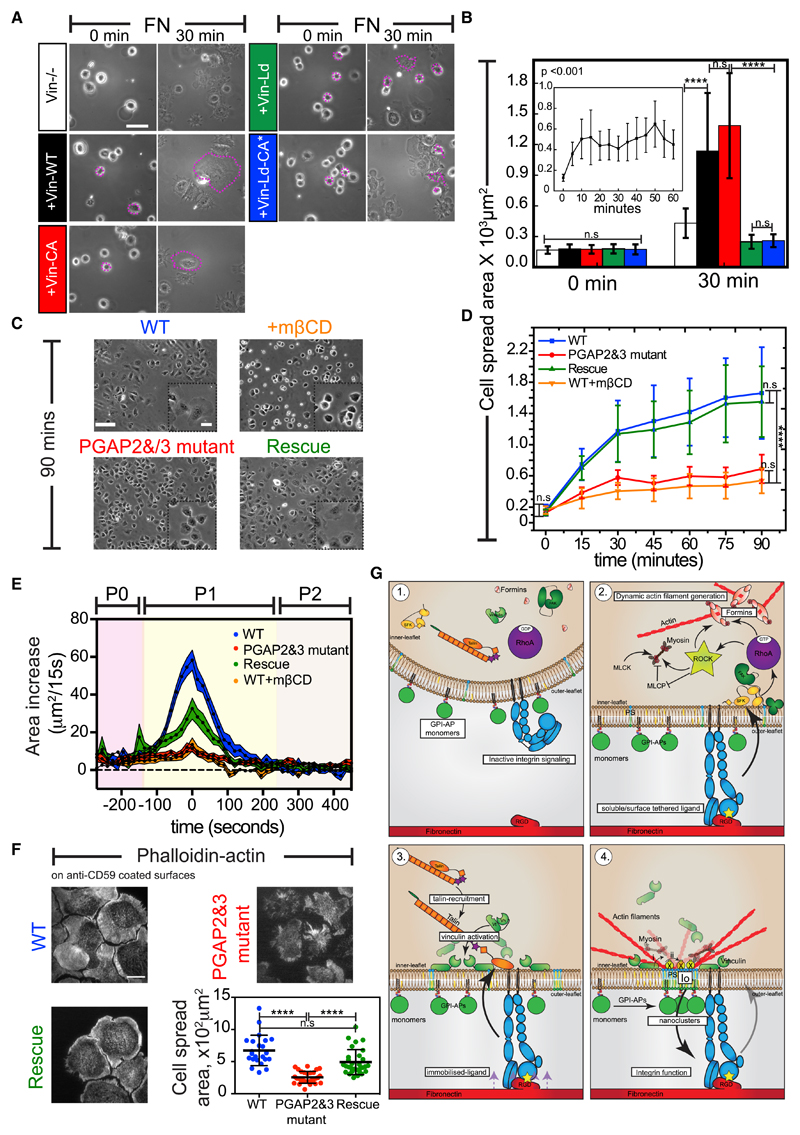
Activity-Generated GPI-AP Nanoclusters Are Necessary for Efficient Cell Spreading on FN (A and B) Phase-contrast images (A) of Vin^−/−^ cells without or with transient transfection of the indicated GFP-Vin constructs after seeding on FN for the indicated time. Magenta dotted lines outline the transfected cells. Bar graphs (B) show the mean spread area. Inset depicts the cell-spreading profile of Vin^−/−^ cells on FN. Scale bar, 100 μm. (C and D) Images of WT (blue), PGAP2 and PGAP3 mutant (red), rescued cells (green), or WT cells pre-treated with 10 mM mβCD after seeding on FN for the indicated time. The corresponding mean cell-spread area profile is shown in (D). Scale bar, 100 μm and 25 μm (inset). (E) Plot of mean cell-spread-area change, between two consecutive frames of 15 s, as a function of spreading time on FN for the indicated cell lines. Shaded error bar represents SEM. (F) Representative phalloidin-actin-stained images and scatter dot plot with the mean whole cell spread area of the indicated cell lines plated on anti-CD59 functionalized glass surfaces. Scale bars, 10 μm. (G) Model for the integrin triggered generation of functional GPI-APs nanoclusters. Refer to Discussion for details. All error bars represent SD except in (E). n.s. p > 0.05, *p ≤ 0.05, **p ≤ 0.01, ***p ≤ 0.001, ****p ≤ 0.0001. Sample size and p values are provided in Table S4. See also Figure S7 and Videos S2 and S3.

We next investigated whether the GPI-AP nanoclusters were being generated to regulate integrin function. Cells that lacked vinculin had aberrant FAs (Thievessen et al., 2013) and exhibited defects in integrin-mediated spreading (Figures 7A and 7B). We hypothesized that some of these defects could be attributed to the inability of these cells to build functional nanoclusters, as had been earlier suggested in studies that documented the effects of cholesterol depletion on the integrin adhesion response (Norman et al., 2010; van Zanten et al., 2009). However, due to the pleiotropic nature of these perturbations, it is difficult to conclude a role of the nanoclusters in the process. To address this, we utilized mutant cells that were deficient in two enzymes, PGAP2 and PGAP3, required for the remodeling of the unsaturated GPI-anchor acyl chains to long saturated chains (Maeda et al., 2007). This defect resulted in their inability to make GPI-AP nanoclusters, without an ensuing defect in the actomyosin-based nanoclustering machinery (Raghupathy et al., 2015). Therefore, we utilized this cell-based system to study the functional role of GPI-AP nanoclustering in the regulation of integrin-mediated responses.

In contrast to their wild-type (WT) counterparts, the mutant cells failed to exhibit a decrease in the anisotropy of GFP-GPI on FN, despite having intact cell-surface levels of the protein, and this defect was reversed by restoring the activities of the PGAP2 and PGAP3 enzymes (Figures S7A and S7B). Importantly, the mutant cells also exhibited a defective cell-spreading response on FN and lacked the characteristic P1 phase that was observed in WT or rescue cells (Figures 7C–7E; Video S2). The mutant cells lacked a protrusive lamellipodia and possess fewer smaller adhesions (Figures S7G and S7H) and exhibited bleb-based cell spreading on FN (Video S3). This defect was not due to defects in integrin activation on FN, since antibodies that bind to either active or in-active conformations of the β1-integrins bound equivalently to both the mutant and rescued cells (Figure S7D). Thus, the enhanced GPI-AP nanoclustering on FN observed at the P0-P1 transition point (Figure 2C) was essential for the progression into the P1 phase of cell spreading.

To further corroborate the functional role of GPI-AP clustering in the integrin-mediated response, we monitored cell spreading on antibody-coated surfaces (Figure S7C). The antibodies bound to and ectopically clustered GPI-APs (FR-GPI or CD59) expressed on the cell surface and initiated robust cell spreading (Figures 7F and S7I). The cells that spread on antibody-coated surfaces exhibited a diffuse cytosolic distribution of paxillin, in contrast to cells plated on FN or cRGD where paxillin-containing adhesions were observed (Figure S7J). Moreover, the antibody-induced cell-spreading response was not observed when cells overexpressed a folate receptor transmembrane (FR-TM; Figure S7I) or when GPI-AP was cross-linked in PGAP2 and PGAP3 mutant cells (Figure 7F). Thus, the antibody-induced cell-spreading response occurred in the absence of integrin ligands and associated signaling response and required the presence of an appropriately remodeled GPI anchor.

Several proteins that either create or reside within liquid ordered (*lo*)-like regions on the cell membrane have been implicated in the process of cells spreading and migration (Moissoglu et al., 2014; Navarro-Lérida et al., 2012). We had previously shown using atomistic simulations that the immobilized sites where long-saturated-acyl-chain-containing GPI anchors couple with PS at the inner leaflet have an *lo*-like character (Raghupathy et al., 2015). Therefore, we tested whether the lack of GPI-AP nanoclusters in the PGAP2 and PGAP3 mutants could also lead to a global disruption of ordered domains that can explain some of the observed defects in cell spreading. For this, we utilized general polarization (GP)-based measurements of the polarity-sensitive dye, Laurdan (6-lauryl-2-dimethylamino-napthalene), as a reporter of membrane order (Owen et al., 2011). We found that the mutant cells exhibited a lower GP value (Figures S7K and S7L) compared to WT cells. The decrease in the GP value was similar to that observed when membrane cholesterol was depleted in WT cells using mβCD and was fully restored in the rescue cells (Figures S7K and S7L). To further confirm that the loss of *lo* domains was due to a specific defect in GPI-AP nanoclustering, we assessed the levels of filipin and compared the mass-spectra profiles from blebs extracted from these cells. We did not find any significant difference either in the levels of free membrane cholesterol or in the phospholipid profiles between the mutant and WT or rescue cell types (Figures S7E and S7F; Table S2). Together, these results suggest that the lack of GPI-AP nanoclustering specifically contributes to the loss of *lo* domains at the cell surface.

Finally, we studied the role of the GPI-AP nanoclustering in mediating long-term integrin-dependent functions in the cell. In addition to its effects in integrin-based cell spreading, we assessed the role of GPI-AP nanodomain formation in regulating integrin-dependent cell migration. Compared to WT cells and rescue cells, the PGAP2 and PGAP3 mutants exhibited a significant delay in their ability to migrate in a scratch assay (Figures S7M and S7N). Thus, the ability to induce nanodomains of GPI-APs is required for efficient cell spreading and migration, key hallmarks of integrin activation.

## Discussion

Our results, using both chemical and genetic perturbation approaches, show how nanoclustering of GPI-APs is initiated via a signaling cascade triggered by β1-integrin receptor binding to its bona fide RGD-containing ligands (see model in Figure 7G). While ligand engagement results in the activation of RhoA GTPase downstream of SFK and FAK, molecules including ILK and kindlin kinases might additionally be involved in the process (Calderwood et al., 2013). Downstream of RhoA, the activity of the linear actin nucleator formins, and in particular FHOD1 and not the branched actin nucleator Arp2/3 complex, is required for GPI-AP nanoclustering. RhoA activates ROCK which can first activate myosin function (Amano et al., 1996; Kimura et al., 1996) and second phosphorylate the C-terminal serine and threonine residues in the DAD region of the formin FHOD1, relieving its autoinhibition (Takeya et al., 2008) and together facilitate the generation of the dynamic acto-myosin machinery required for GPI-AP nanoclustering.

Although src, FAK, and the formin FHOD1 are recruited to the nascent integrin clusters that form when RGD ligands are mobile on SLBs (Yu et al., 2011), GPI-AP nanoclustering requires the immobilization of ligand. Binding to RGD ligands immobilized on glass or transiently in SLBs prepared on nanopatterns results in the application of traction force on the integrin receptors (Yu et al., 2011), causing the stretching of talin and activation and recruitment of vinculin. Vinculin activation is a crucial step in the integrin-mediated mechano-chemical modulation of GPI-AP nanoclustering. Consistent with this, chronically activated vinculin is sufficient to restore GPI-AP nanoclustering even when mobile integrin ligands were presented on SLBs.

Transmembrane proteins with actin-binding motifs directly associate with the dynamic acto-myosin machinery (Gowrishankar et al., 2012) and hence do not require additional adapters for their nanoclustering. In contrast, GPI-APs present at the outer leaflet engage in transbilayer interactions with long acyl-chain-containing PS lipids at the inner leaflet (Raghupathy et al., 2015), and additional membrane associated adapters that couple PS with the dynamic actin-myosin machinery are required for facilitating GPI-AP nanoclustering. Vinculin could serves as this link, and the failure of the lipid or actin-binding mutants to restore GPI-AP nanoclustering in Vin^−/−^ cells supports this hypothesis. However, vinculin was not found measurably enriched at the membrane outside of FAs, at time points when GPI-AP nanoclustering is restored (Figures S6E and S6F), suggesting that vinculin might only indirectly facilitate this link. Further mechanistic understanding of this linkage remains to be explored.

The physiological relevance of nanoclustering has been difficult to ascertain because of the use of drastic perturbations such as cholesterol removal that is often employed (Kwik et al., 2003). The PGAP2 and PGAP3 mutant cells offer an alternative approach to study this. Due to defects in enzymes that remodel the GPI anchor, these mutant cells lack the ability to efficiently cluster GPI-APs on FN, despite having normal surface levels of GPI-APs (Jaensch et al., 2014; Maeda et al., 2007) and possessing all the machinery for clustering (Raghupathy et al., 2015). We observed that, although these mutants support ligand-dependent integrin-activation and FA formation, they do not generate a protrusive lamellipodia, lack small nascent adhesions at the cell periphery, and instead make large FAs when freshly plated on FN. They also exhibit a delayed migratory response in a scratch assay. Altogether, this implicates a broader role for GPI-AP nanoclustering in the regulation of integrin turnover.

Why does signaling via integrin-ligation activate the local construction of GPI-AP nanoclusters? One explanation is related to the understanding that GPI-AP nanoclusters form local *lo* nanodomains (Raghupathy et al., 2015). Consistent with this, we find that the GPI-AP nanoclustering-defective PGAP2 and PGAP3 cells also exhibit a lower *lo* membrane characteristic. The generation of *lo*-like domains around integrin-enriched FA sites and the re-localization of several lipid raft components during cell detachment have been previously described (Gaus et al., 2006; Norambuena and Schwartz, 2011). Our results suggest a mechanism for the generation of GPI-AP nanocluster-rich, *lo*-like membrane regions in effecting integrin responses. While a relationship of the GPI-AP nanoclusters to the larger-scale *lo* domains around the membrane receptor is to be expected, how these larger-scale domains are built remains open and is a subject of further investigation.

Lipid modifications such as palmitoylation enable molecules to partition into such locally generated *lo* microenvironments (Lorent and Levental, 2015). The palmitoylation of fyn kinase is implicated in rigidity-sensing mechanisms required for the P1-phase of cell spreading (Kostic and Sheetz, 2006). Integrin-triggered lamellipodial protrusive activity that depends on palmitoylated Rac1 and the signaling activities of the SFK and FAK kinases are also restricted to *lo* domains (Moissoglu et al., 2014; Navarro-Lérida et al., 2012; Seong et al., 2011). Cross-linking of outer-leaflet GPI-APs is sufficient to accumulate SFK kinases at the inner leaflet (Harder et al., 1998; Stefanová et al., 1991; Suzuki et al., 2007). Consistent with this, we find that cross-linking GPI-APs with an intact anchor using surface-bound antibodies is sufficient to activate cell spreading even in the absence of integrin signaling.

A second explanation stems from the observation that GPI-APs such as Thy-1 and uPAR have been shown to bind to and modulate integrin conformation and mechanotransduction (Fiore et al., 2015; Wei et al., 2005), and *lo-like* nanodomains might serve as an additional tier to localize and regulate such interactions within the cell.

In conclusion, this study uncovers the molecular mechanism for the control of an active actin-membrane composite, wherein the fluid membrane is inextricably coupled to the cortical actin substructure beneath (Köster and Mayor, 2016). The functioning of this composite implies regulation (Gowrishankar et al., 2012), and here we provide evidence for such an active element, exemplified by the integrin receptor family, that is capable of regulating elements within this composite. Since the activation of RhoA and vinculin is a pivotal feature downstream of other signaling receptors including cadherins (Hazan et al., 1997; Olson and Nordheim, 2010), our results suggest a generalizable picture of how GPI-AP-enriched nanodomains may be created and deployed. The resultant membrane domains that ensue will serve as allosteric modulators of the output of the signaling system that generates it (Chaudhuri et al., 2011; Harding and Hancock, 2008) and regulators of receptor cross-talk (Mattila et al., 2016). The generation of a nanocluster-enriched microenvironment created by the mechano-chemical-gated signaling mechanism could serve to coordinate a number of important effector cascades at the PM. This will naturally allow the cell to integrate information that is encoded primarily in the chemical composition of its membrane bilayer with mechanical inputs.

## Star★Methods

**Table:** Key Resources Table

REAGENT or RESOURCE	SOURCE	IDENTIFIER
Antibodies		
Diap1(mDia1) Antibody	Cell Signaling Technology	Cat# 5486; RRID: AB_10828440
FHOD1 Antibody	ECM Biosciences	Cat# FM3521; RRID: AB_2104508
Integrin β1 Antibody,non-function perturbing, K20	Santa Cruz	Cat# sc-18887; RRID: AB_627006
Integrin β1 Antibody,blocking, 4B4	Beckman Coulter	Cat# 6603113; RRID: AB_10638675
Integrin β1 Antibody, activated, clone HUTS-4	Merck	Cat# MAB2079Z; RRID: AB_2233964
Transferin Antibody,OKT9	Purified from hybridoma cells procured from National Centre for Cell Science,Pune,India	NA
FR Antibody, MOv 19	Dr Silvana Canevari (Fondazione IRCCS Istituto Nazionale dei tumori di Milano,Italy)	NA
CD59 Antibody, MEM-43	Abcam	Cat# ab9182; RRID: AB_307053
Talin Antibody, Clone 8D4	Sigma Aldrich	Cat# SAB4200694
Vinculin Antibody, Clone SPM227	Abcam	Cat# ab18058; RRID: AB_444215
Paxillin Antibody	BD Transduction Labs	Cat# 610051; RRID: AB_397463
AffiniPure Donkey Anti-Mouse IgG (H+L)	Jackson ImmunoResearch Laboratories, Inc.	Cat# 715-005-150; RRID: AB_2340758
β-actin Antibody	Sigma Aldrich	Cat# A5060; RRID: AB_476738
Peroxidase AffiniPure Donkey Anti-Rabbit IgG (H+L)	Jackson ImmunoResearch Laboratories, Inc.	Cat# 711-035-152; RRID: AB_10015282
Peroxidase-AffiniPure Goat Anti-Mouse IgG (H+L)	Jackson ImmunoResearch Laboratories, Inc.	Cat# 115-035-003; RRID: AB_10015289
CaptureSelect Biotin Anti-IgG-Fc (Multi-species) Conjugate	Thermo Scientific	Cat# 7102852100
Chemicals, Peptides, and Recombinant Proteins		
Human Plasma Fibronectin Purified Protein	Merck (Sigma Aldrich)	Cat# FC010
H-Gly-Pen-Gly-Arg-Gly-Asp-Ser-Pro-Cys-Ala-OH trifluoroacetate salt (Disulfide bond between Pen^2^ and Cys^9^)	Bachem	Cat# H-3964
SFK Inhibitor; PP2	Calbiochem (Merck)	Cat# 529573
FAK Inhibitor; PF 573228	Sigma Aldrich	Cat# PZ0117
C3 exoenzyme; Rho Inhibitor I	Cytoskeleton, Inc.	Cat# CT04
Rho Activator II	Cytoskeleton, Inc.	Cat# CN03
Formin Inhibitor; SMIFH2	Sigma Aldrich	Cat# S4826
Arp2/3 Inhibitor; CK666	Sigma Aldrich	Cat# SML0006
ROCK Inhibitor; Y-27632 dihydrochloride	Sigma Aldrich	Cat# Y0503
MLCK Inhibitor; ML-7	Sigma Aldrich	Cat# I2764
18:1 (Δ9-Cis) PC (DOPC) 1,2-dioleoyl-sn-glycero-3-phosphocholine	Avanti Polar Lipids, Inc.	Cat# 850375
16:0 Biotinyl Cap PE 1,2-dipalmitoyl-sn-glycero-3-phosphoethanolamine-N-(cap biotinyl) (sodium salt)	Avanti Polar Lipids, Inc.	Cat# 870277
NeutrAvidin Protein, DyLight 650	Invitrogen (Thermo Fisher Scientific)	Cat# 84607
Avidin, NeutrAvidin Biotin-binding Protein	Invitrogen (Thermo Fisher Scientific)	Cat# A2666
Cyclo[Arg-Gly-Asp-D-Phe-Lys(Biotin-PEG-PEG)]	Peptides International	Cat# PCI-3697-PI
Laminin from Engelbreth-Holm-Swarm murine sarcoma basement membrane	Sigma Aldrich	Cat# L2020
Collagen I Rat Protein, Tail	GIBCO (Thermo Fisher Scientific)	Cat# A1048301
Poly-L-lysine solution, 0.01%	Sigma Aldrich	Cat# P4707
Vitronectin from Human Plasma	Sigma Aldrich	Cat# V9881
Methyl-β-cyclodextrin	Sigma Aldrich	Cat# C4555
FuGENE® 6 Transfection Reagent, For plasmid DNA transfections	Promega	Cat# E2692
HiPerFect Transfection Reagent, For siRNA transfections	QIAGEN	Cat# 301704
Diaphanous (mDia)-related Formin Agonist, IMM01	Merck Millipore	Cat# 509583
6-Dodecanoyl-N,N-dimethyl-2-naphthylamine	Sigma Aldrich	Cat# 40227
Filipin III from *Streptomyces filipinensis*	Sigma Aldrich	Cat# F4767
Alexa Fluor 488 phalloidin	Invitrogen (Thermo Fisher Scientific)	Cat# A12379
Alexa Fluor 568 phalloidin	Invitrogen (Thermo Fisher Scientific)	Cat# A12380
PLL(20)-g[3.5]-PEG(2)/PEG(3.4)biotin 50%	SuSoS	Cat# PLL(20)-g[3.5]-PEG(2)/PEG(3.4) biotin 50%
Proaerolysin	Protein Technology Core (C-CAMP,Bangalore,India)	N/A
Alexa 488- conjugated FLAER	Protox Biotech	Cat# FL1S
Hellmanex III	Hellma Analystics (Sigma Aldrich)	Cat# Z805939
Experimental Models: Cell Lines		
GG8;CHO-K1 cells (TRVb-1), devoid of transferrin receptor (TfR) were stably transfected with human TfR and EGFP-GPI	Satyajit Mayor (Sabharanjak et al., 2002)	N/A
U2OS; Human Osetosarcoma cells	Clare Waterman (NHLBI,NIH,USA) (Case and Waterman, 2011)	N/A
U2OS GG; U2OS cells stably transfected with GFP-GPI	This paper	N/A
Vin−/− MEFs;Mouse embryonic fibroblasts deficient in vinculin	Daniel Rösel (Charles University in Prague, Czech Republic) (Janoštiak et al., 2014)	N/A
GD3S-C37;3B2A cells (CHO cells stably expressing CD59 and DAF) stably expressing GD3 synthase	Taroh Kinoshita (Osaka University, Japan)(Tashima et al., 2006)	N/A
C84 DM2&3-C2;PGAP2/3 double mutant cell line stably expressing CD59 and DAF	Taroh Kinoshita (Osaka University, Japan)(Maeda et al., 2007)	N/A
C84 DM2&3-C2 + pMSCV-rPGAP2-zeo-hPGAP3;PGAP2/3 double mutant cells expressing PGAP2 and PGAP3	Taroh Kinoshita (Osaka University, Japan) (Maeda et al., 2007)	N/A
Talin 1 deficient MEFs; Mouse embryonic fibroblasts deficient in talin 1	Mike Sheetz (Mechanobiology Institute, Singapore) (Zhang et al., 2008)	N/A
CHOB2-α5-GFP;CHOB2 (that lack endogenous a5integrin) cells stably expressing GFP-a5 integrin	Alan F Horwitz (University of Virginia,USA) (Laukaitis et al., 2001)	N/A
FAK −/− MEFs; Mouse embryonic fibroblasts that have null mutations in FAK and p53 gene derived from E 8.0 embryo	ATCC (llić et al., 1995)	Cat# CRL-2644; RRID: CVCL_8954
FAK +/+ MEFs;Mouse embryonic fibroblasts in which there is mutation in only the p53 gene and used as a control for the FAK−/− line	ATCC (llić et al., 1995)	Cat# CRL-2645; RRID: CVCL_8955
MYG1;m-YFP GPI expressing CHO cells	Satyajit Mayor (Suvrajit Saha PhD Thesis)	N/A
Oligonucleotides		
Primers used to generate the GFP-Vinculin Ld CA	See STAR Methods	N/A
Primers used to generate the GFP-Vinculin Ld construct	See STAR Methods	N/A
Primers used to generate the Lact C2 AAA-Ezrin AFBD-YFP construct	See STAR Methods	N/A
ON-TARGETplus Non-targeting siRNA	Dharmacon	Cat# D-001810-01-05
siGENOME Human FHOD1 siRNA SMART Pool	Dharmacon	Cat# M-013709-01-0005
siGENOME Human DIAPH1 siRNA SMART Pool	Dharmacon	Cat# M-010347-02-0005
Primers used to generate the mRuby2-GPI construct	See STAR Methods	N/A
Recombinant DNA		
Talin 2 shRNA	Mike Sheetz (Mechanobiology Institute, Singapore) (Zhang et al., 2008)	N/A
GFP-Vinculin WT;Full length	Clare Waterman (NHLBI,NIH,USA) (Thievessen et al., 2013)	N/A
mCherry-Vinculin WT;Full length	Clare Waterman (NHLBI,NIH,USA) (Pasapera et al., 2010)	N/A
GFP-Vinculin CA;constitutively active	Clare Waterman (NHLBI,NIH,USA) (Case et al., 2015)	N/A
GFP-Vinculin A50I;Talin non-binding	Clare Waterman (NHLBI,NIH,USA) (Case et al., 2015)	N/A
GFP-Vinculin A50I CA;Talin non-binding constitutively active	Clare Waterman (NHLBI,NIH,USA) (Case et al., 2015)	N/A
GFP-Vinculin Ld; Lipid non-binding	This Paper	N/A
GFP-Vinculin Ld CA; Lipid non-binding constiutively active	This Paper	N/A
GFP-Vinculin AB1; Actin non-binding	Clare Waterman (NHLBI,NIH,USA) (Case et al., 2015)	N/A
GFP-Vinculin AB1 CA; Actin non-binding constitutively active	Clare Waterman (NHLBI,NIH,USA) (Case et al., 2015)	N/A
GFP-Vinculin head; head domain of vinculin	Clare Waterman (NHLBI,NIH,USA) (Case et al., 2015)	N/A
Lact C2 Ezrin AFBD-YFP	Protein Technology Core (C-CAMP,Bangalore,India) (Raghupathy et al., 2015)	N/A
Lact C2-AAA Ezrin AFBD-YFP	This Paper	N/A
GFP-Vinculin AB2; Actin non-binding constitutively active	Clare Waterman (NHLBI,NIH,USA) (Case et al., 2015)	N/A
mRFP-tH	John F.Hancock (University of Queensland,Australia) (Plowman et al.,2005)	N/A
mRuby2-GPI	This Paper	N/A
pcDNA3-mRuby2	Michael Lin (Stanford University, USA) (Lam et al., 2012)	Addgene plasmid # 40260; http://addgene.org/40260; RRID: Addgene_40260)
Software and Algorithms		
MATLAB, Image processing toolbox	Mathworks Inc.	https://www.mathworks.com/products/matlab.html
Fiji Image analysis software	Schindelin et al., 2012	https://fiji.sc
GraphPad Prism 7 software	GraphPad	https://www.graphpad.com/scientific-software/prism/

### Contact for Reagent and Resource Sharing

Further information and requests for resources and reagents should be addressed to and will be fulfilled by the Lead Contact, Satyajit Mayor (mayor@ncbs.res.in).

### Experimental Model and Subject Details

#### Culture and Maintenance of Cells

Chinese Hamster Ovary (CHO) cells stably expressing EGFP-GPI were maintained in Ham’s F12 media (HiMedia) supplemented with 10% heat inactivated FBS and Penicillin-Streptomycin-Glutamine (PSG) [Hereby referred to as complete media] and in the presence of 200 μg/ml G418 and 100 μg/ml Hygromycin B (Sigma Aldrich). Human osteosarcoma derived U2OS cells stably expressing mEGFP-GPI were maintained in McCoy’s 5A media (HiMedia) supplemented with 300 μg/ml G418. GD3S-C37 WT CHO cells stably expressing the GPI-APs CD59 and DAF (CD55) were maintained in Ham’s-F12 complete media supplemented with 600 μg/ml G418, 6 μg/ml Puromycin. The PGAP2/3 double mutant were maintained in medium supplemented with 600 μg/ml G418, 6 μg/ml Puromycin and 6 μg/ml Blasticidin (as described in Maeda et al., 2007 and Tashima et al., 2006). Additionally, for the maintenance of the PGAP2/3 mutants exogenously expressing PGAP2 and PGAP3 enzymes (Rescue cell line; pMSCV-rPGAP2-zeo-hPGAP3), 250 μg/ml Zeocin was used as selection antibiotic in the media. FR-TM-Ez-AFBD and FR-TM-Ez-AFBD* (RA mutant) cell line was generated as described previously (Gowrishankar et al., 2012) and was maintained in Ham’s F12 complete media. Vinculin and Talin 1 deficient mouse embryonic fibroblasts (MEF) were maintained in DMEM, High glucose (HiMedia) supplemented with 10% FBS and PSG (Invitrogen). M1 buffer (150 mM NaCl, 20 mM HEPES, 5 mM KCl, 1mM CaCl_2_ and 1mM MgCl_2_-pH 7.4) with 1 mg/ml Glucose for CHO cells and 4.5g/L Glucose for MEFs was used for live imaging.

#### Plasmid constructs

The cDNA encoding LactC2-Ez AFBD-YFP was obtained from the Protein Technology Core at C-CAMP (Raghupathy et al., 2015). Vin-head construct was generated by inserting vinculin head (1-821 amino acids) into EGFP-C3 vector using ECOR1 restriction enzyme (New England Biolabs) and mRuby2-GPI was generated by replacing the GFP portion of the GFP-GPI plasmid (pJB20 back-bone) with mRuby2 from the pcDNA3-mRuby2 plasmid generated in Michael Lin’s laboratory, Stanford University, USA using Gibson assembly (New England Biolabs).

The primer pair used to amplify the backbone:

**Table:** 

**FP:**	5′-GCT GCA GCC ATG AGT GGG G-3′
**RP:**	5′-CAG GTC CTC CTC GCT TAT TAG TTT TTG-3′

The primer pair used to amplify the mRuby2 portion:

**Table:** 

**FP:**	5′-AAC TAA TAA GCG AGG AGG ACC TGA TGG TGT CTA AGG GCG AAG-3′
**RP:**	5′-GCC CCA CTC ATG GCT GCA GCC TTG TAC AGC TCG TCC ATC-3′

The Vin-Ld-CA*, Vin-Ld and LactC2-AAA-Ez AFBD constructs were generated by site directed mutagenesis as described below:

To generate the vinculin construct that is constitutively active (T12) with Ld mutation Vin-Ld-CA*, amplicons using FP1 and RP1 were generated from the pmEGFP-Vinculin13 backbone [Wild-type]. The nucleotide sequences used to generate the mutations are underlined.

**Table:** 

**FP1:**	5′-CAG GAT ATC GCC CAG GCC TCT GAT GAG GTG ACG CAG TTG GCC CAG GAG GTT GCC AAG CAG TGC ACA GCT GCG GCG ATT AGA ACC AAT CTC TTA CAG GTA TGC GAG-3′
**RP1:**	5′-GGC ACA CTG AAT AAG TGC CCG CTT-3′

Sequence verified clones were then used for generating subsequent mutations in a stepwise fashion with each step involving a PCR with corresponding primer pairs, colony selection and sequencing to confirm the mutations.

**Table:** 

**FP2:**	5′ -TGC ACA GCT GCG GCG ATT GCA ACC AAT CTC TTA CAG GTA-3′
**RP2:**	5′ -TAC CTG TAA GAG ATT GGT TGC AAT CGC CGC AGC TGT GCA-3′
**FP3:**	5′ -ACT CTG CGC TGG GTC CAA AAG ACT CCC TGG TAC-3′
**RP3:**	5′ -GTA CCA GGG AGT CTT TTG GAC CCA GCG CAG AGT-3′
**FP4:**	5′ -CTG CGC TGG GTC CAA CAG ACT CCC TGG TAC CAG-3′
**RP4:**	5′ -CTG GTA CCA GGG AGT CTG TTG GAC CCA GCG CAG-3′

Likewise, we used the following set of primers to generate the Vin-Ld construct that has a deficiency in lipid binding:

**Table:** 

**FP1:**	5′ CAG GAT ATC GCC CAG GCC TCT GAT GAG GTG ACG CAG TTG GCC CAG GAG GTT GCC AAG CAG TGC ACA GAT AAG-3′
**RP1:**	5′ -GGC ACA CTG AAT AAG TGC CCG CTT-3′
**FP2:**	5′ -ACT CTG CGC TGG GTC CAA AAG ACT CCC TGG TAC-3′
**RP2:**	5′ -GTA CCA GGG AGT CTT TTG GAC CCA GCG CAG AGT-3′
**FP3:**	5′ -CTG CGC TGG GTC CAA CAG ACT CCC TGG TAC CAG-3′
**RP3:**	5′ -CTG GTA CCA GGG AGT CTG TTG GAC CCA GCG CAG-3′

For the **LactC2-AAA-Ez AFBD** construct we used a single primer set to generate the W26A,W33A and F34A mutation using the LactC2-Ezrin AFBD YFP as a template. Wild-type codons are represented within parenthesis and the alanine mutation sequences are underlined.

**Table:** 

**FP1:**	5′-(GGG)GCGCCT GAG TGC CTT TAG CTG (GTT)GCT(TCC)GCC CTACTACGCACGACTGGATAATCA-3′
**RP1:**	: 5′-CAGGTTTTGTAGTAGCTGGAGGCTGTGATCTGCTTGTTGGGGATG-3′

### Method Details

#### GPI analog incorporation

GPI analogs were incorporated into cell membranes either by γ–CD (Koivusalo et al., 2007) or by lipofectamine method (Raghupathy et al., 2015), as described earlier. Briefly, cells were incubated with lipid-lipofectamine complexes (1 μM lipofectamine-GlcNPI-NBD and 0.5 μM of lipofectamine-C6-NBD-SM complexes; concentrations adjusted so as to get the same extent of incorporation for both probes) for 30 min at 37°C, followed by de-adhering and re-plating cells onto FN coated dishes. This was done to get rid of lipid sticking on the FN coated coverslip. However, during the process of re-plating at 37°C, some fraction of the lipids at the plasma membrane were internalized into endosomes. These were visible as bright punctate structures and were excluded from subsequent analysis. Care is also taken that the addition of lipofectamine complexes as such does not alter membrane properties. For a detailed characterization of these methods of lipid incorporation please refer to Riya Raghupathy’s Doctoral thesis (http://shodhganga.inflibnet.ac.in:8080/jspui/handle/10603/77067).

#### Purification of pro-aerolysin

The inactive toxin pro-aerolysin was purified from 500 mL of bacterial culture [compilation of the protocol provided by Suzanne Salvi & Loan Lacovache from Van der Goot laboratory, and performed at PTC, C-CAMP (Fivaz et al., 2002)]. Post bacterial transformation a single colony was picked and inoculated into 100 mL of Luria Broth (LB) culture supplemented with Ampicillin and this was incubated overnight at 37°C in a shaker at 250rpm. After achieving an OD_600_ of ~4-5, the culture was diluted by a factor of 1:20 (10mL for every 500 mL of the media) and incubated at 37°C in an incubator shaker at an rpm of 250 until the OD_600_ reached ≈ 0.5-0.6 (time required for this is ~2.5 hours). This was followed by induction using 1mM IPTG. This was further incubated for 3-5 hours at 26°C at 250rpm.

The cells were harvested by centrifugation at 4000 rpm for 30 minutes at 4°C. The cells were re-suspended in T buffer (0.1M Tris-HCl, pH 8.0, 18% (m/v) sucrose) and EDTA was added to obtain a final concentration of 5mM. After vortexing the sample was incubated for 5 minutes on ice. Lysozyme was added to a final concentration of 0.2 mg/mL followed by vortexing and incubation at 4°C for 30 minutes (this incubation was done by gentle agitation for the entire incubation time). The periplasmic fraction was harvested by centrifugation at 1000 rpm for 30 min at 4°C and the supernatant was collected. 1,10-phenanthroline was added to a final concentration of 1mM.

The periplasmic fraction was dialyzed into Buffer A to completely remove EDTA. The total volume of the buffer used for dialysis is 1L and the dialysis was performed three times. The aggregates were removed after centrifugation at 25,000 rpm for 1 hour at 4°C. For purification the sample was loaded onto a HisPrepFF 16/10. His-Tag purification protocol was run on AKTA with a linear gradient from Buffer A to Buffer B and the desired product eluted at ≈50% buffer B. The fraction was collected and dialyzed into storage buffer (20mM MES, 150mM NaCl, pH 5.5). The product was aliquoted and stored at –80°C for future use.

#### Coating of glass coverslips with extracellular matrix (ECM) proteins

No 1 glass (130-170 μm thickness) coverslip bottom dishes were UV sterilized and 200 μL of 10 μg/ml (diluted in 1XPBS) human plasma fibronectin (Merck Millipore) solution (or 20 μg/ml EHS laminin,10 μg/ml human plasma vitronectin or 50 μg/ml collagen-1 diluted in 20mM acetic acid) was added to the center of each dish and incubated at 37°C for 2 h or 4°C overnight. The coating density has been shown to linearly depend on the FN solution concentration at the indicated range used here (Gupton and Waterman-Storer, 2006). Poly-L-lysine coating was done as per the protocol described in Case and Waterman (2011)

Prior to plating cells, the dishes were washed with 1XPBS thrice to remove any unbound FN that would remain, and additionally once with 1XM1-Glucose medium to replace the PBS, taking care not to dry the coated protein in the process.

#### Preparation of cRGD and antibody functionalized glass substrates

No 1 glass coverslips (Warner Instruments/Harvard Apparatus) were cleaned by sonication for 15 mins in 100% EtOH and rinsed several times in de-ionized (DI) water and activated with deep-UV (~185 nm) for 10 mins in a stream of oxygen. The cleaned and activated coverslips were dried in a stream of N2 gas and dropped onto 30 μl of 1mg/ml PLL(20)-g[3.5]-PEG(2)/PEG(3.4)-biotin 50% (SuSoS) (~30μgs) and incubated for 30 mins at RT. The polymer is composed of poly-l-lysine (PLL) (MW: 24700 g/mol) backbone onto which poly(ethylene glycol) (PEG-O-CH3) (MW: 2001 g/mol) and PEG-biotin (51%) (MW: 3687 g/mol) is grafted. The coverslips were then assembled onto Attofluor imaging chambers (Invitrogen) submerged in a water trough. The water in the chamber was then replaced with 1XPBS by repeated exchanges and subsequently incubated with 1%BSA (Sigma Aldrich) (in 1XPBS) for 30 mins at RT. Avidin,Neutravidin biotin-binding protein (MW:60 KDa; Invitrogen,Thermo Fisher Scientific) was added to the center of each dish to a final amount of 15 μgs and incubated for exactly 10mins at RT. The excess neutravidin was washed off and varying amounts of biotinylated cRGD (Cyclo[Arg-Gly-Asp-D-Phe-Lys(Biotin-PEG-PEG)]; MW:1120.30 g/mol; Peptides International) (0,5,10 and 15 μgs that corresponds to 0,4.5 μM, 9 μM and 13.4 μM respectively) was added to the center of each chamber and incubated for 30-45 mins at RT. Unbound cRGD was washed thoroughly with 1XPBS. Just before adding cells, the PBS was replaced with 1XM1-Glucose.

For the antibody cross-linking experiments, the same protocol was followed. But instead of biotinylated cRGD, 5 μg of CaptureSelect Biotin Anti-IgG-Fc conjugate (Thermo Fisher Scientific) was added for 30 mins followed by several washes and additional incubation with 5μg of anti-FR (MOv19) (Dr Silvana Canevari) or anti-CD59 antibody (Abcam). Extreme care is taken while mixing, to avoid any inhomogeneity in ligand coating that can lead to variability in cell spreading.

#### Preparation of cRGD functionalized Supported Lipid Bilayers (SLBs)

Supported lipid bilayers functionalized with cRGD was prepared based on the published protocol (Yu et al., 2011) and is outlined below:

#### a) Preparation of small unilamellar vesicles (SUVs)

1,2-dioleoyl-sn-glycero-3-phosphocholine (DOPC) and 1,2-dipalmitoyl-sn-glycero-3-phosphoethanolamine-N-(cap biotinyl) (16:0 biotinyl-Cap-PE) lipids were purchased from Avanti Polar Lipids (Alabaster, AL, USA). Lipids with a desired composition were mixed in chloroform, and subsequently dried in a stream of nitrogen gas. The mixed lipids were left overnight in a desiccator and were then subsequently hydrated with 2 mL of DI water. Small lipid vesicles, ~100 nm in diameter, were made by repeated freeze (liquid N2)/thaw (42°C) cycles followed by probe-sonication (9.9 s pulse; 3.3 s interval for 10 cycles at 30% max amplitude) in an ice bath, and then centrifuged at 20000 x g for 2 hr. ~1 mL of supernatant solution of small lipid vesicles was collected in fresh tubes and stored at 4°C after layering the tubes with argon gas.

#### b) Preparation of cRGD functionalized supported lipid bilayers (SLBs)

Before membrane deposition, glass substrates (No 1 glass coverslips 25 mm round; Warner Instruments/Harvard Apparatus) were cleaned with 1% Hellmanex III solution (Hellma Analytics) at 60°C in a bath sonicator for 15 mins and rinsed at least five times with excess of DI water. The substrates were then treated with 2N NaOH for 5 mins in the bath sonicator and washed five times in DI water. Plain glass substrates were activated by exposure to deep-UV (~185 nm) in an enclosed container for 10 min with a stream of oxygen, rinsed with 100 mL DI water five times, and dried under a clean N2 gas stream. The lipids (9 parts of 100 mol% DOPC SUVs and 1 part of 2 mol% of biotinyl-Cap-PE: 98mol% of DOPC SUVs) were mixed (to get a final 0.2 mol% biotinyl-Cap-PE) in half its volume of 2xPBS, and 30 μls of this mix was added onto the center of a plastic 35mm dish (Nunc) and the cleaned glass coverslips were dropped onto this for the self-assembly processes. Excess lipid vesicles were removed by immersing the entire dish into a room temperature DI water trough and the lipid-coated glass substrate coated with SLB was then assembled onto a Attofluor imaging chamber (Invitrogen) within the water bath. After assembly, supported lipid membranes in the chamber were always maintained under aqueous conditions. The DI water was slowly exchanged with 1X PBS and all subsequent incubations and washes were performed with 1XPBS. The supported lipid membranes were blocked by incubation of 100 μg/mL of casein for 30 mins at room temperature. DyLight 650 conjugated Neutravidin or dark neutravidin (Invitrogen) [serves as a link between biotinyl-Cap-PE on the bilayer and the biotinylated RGD-peptide] was added onto supported lipid membranes for 30 min in room temperature. Excess neutravidin was removed by serial solvent exchange, 25 mL of PBS in each chamber. Biotinyated cRGD-peptide (Arg-Gly-Asp-D-Phe-Lys(Biotin-PEG-PEG),Mol wt: 1120.30; Peptides International, Louisville, KY, USA) was added to neutravidin-coated supported membranes for 30 min in room temperature. Excess RGD was removed by serial solvent exchange, each chamber, and then finally exchanged with 1XM1-Glucose before addition of cells.

#### c) Preparation of SLBs on chromium nanopatterned surfaces

Nanopatterned chromium metal lines were deposited onto glass coverslips using E-beam lithography as described in Nair et al. (2011) and were provided by the Nano and Micro fabrication core at the Mechanobiology Institute, NUS, Singapore. SLBs were assembled after cleaning the substrates by sonication in 100% EtOH, rinsed in DI water and followed with plasma cleaning. The remainder of the protocol is same as that followed for preparation of continuous SLBs described above.

Serum-starved and cycloheximide treated cells were de-adhered and allowed to recover in suspension for 30 mins at 37°C/5% CO_2_ and then subsequently added onto RGD-functionalized supported membranes and allowed to adhere for 30mins-1hr in 1XM1-1mg/ml Glucose (within 2 hr of preparation of the SLBs). The α5-GFP integrin clusters that form co-localize with the DyLight 650 neutravidin signal (that marks the RGD ligand) and hence can be used as a proxy reporter for RGD clusters (Figure S4I). In the absence of cRGD addition, the cells fail to adhere to the SLB (Yu et al., 2011).

The fluidity of the SLB was assessed by monitoring the fluorescence recovery of DyLight 650 tagged neutravidin in a circular region (~10 μm in diameter) of the SLB post photobleaching. The diffusion coefficient was estimated to be ~0.5 μm^2^/s on the continuous SLBs (outside patterns). The mobile fractions was estimated to be ~90% in the case of continuous SLBs and ~1% for both the SLBs prepared on chromium nanopatterned surfaces and cRGD immobilized on glass with PLL-g-PEG (Table S1). Note that the ROI size chosen for bleaching was much larger than the 2 μm spacing between the chromium lines. The lack of fluorescence recovery on the SLBs prepared on the nanopatterned surface indicates that the chromium grids act as effective barriers to large-scale ligand diffusion (Nair et al., 2011).

#### Preparation of cells for fluorescence experiments

For fluorescence experiments, cells were grown in tissue culture flasks in phenol-free complete media without any selection antibiotics, and transfected with indicated constructs 12-14 hours prior to the experiment using Fugene6 transfection reagent (Promega) as per the manufacturers protocol. Since regular media and FBS contains folic acid, to visualize PLB fluorescence, cells must be grown in folic acid free media supplemented with 10% FBS that has been dialyzed to remove small molecules including folic acid, using 3.5 kDa cutoff snake-skin dialysis tubing (Pierce) submerged in 1XPBS (5 changes of buffer). For the siRNA experiments, cells were transfected with 75 ngs of siRNA (Dharmacon) [dissolved in 1X siRNA buffer (GE Heathcare)] for 72 hours using HiPerfect transfection reagent (QIAGEN) as per the manufacturers protocol.

Prior to the EA-TIRF imaging, cells were serum starved and treated with 50μg/ml cycloheximide for 3 hr (not done for MEFs) to the remove ER and Golgi-associated biosynthetic GFP (or YFP or mRuby2)-GPI pools that contributes to fluorescence (Sabharanjak et al., 2002). Cells were de-adhered using TrypLE (GIBCO, Invitrogen) as per the manufactures protocol. Since fluorescent probes stick to FN, for the experiments involving exogenous addition of fluorescent folic acid analogs in FR-TM-Ezrin-AFBD, FR-TM-Ezrin-RA (FR-TM-Ez-AFBD*) cells and labeling of endogenous GPI-APs in Vin^−/−^ cells, the cells were first labeled with the fluorescent analog of folic acid, PLB (pteroyl lysine conjugated to BODIPY-TMR) or with fluorescent FLAER in 1X-M1-Glucose (Goswami et al., 2008; Raghupathy et al., 2015) for 3 hours at 37°C/5%CO_2_, washed and re-plated back on either glass or FN coated substrates for 1 hr before imaging. Since, during the time of settlement and spreading, there is considerable internalization of the probe, care is taken not to include endocytic structures during the ROI based analysis (Raghupathy et al., 2015).

Pre-treatment with inhibitors was done for 30-60 mins at 37°C in adherent cells followed by de-adhering and cell spreading in the continued presence of the drugs. The concentrations were chosen after performing a dose-response at various concentrations. For PP2 and PF-573 228 mediated inhibition, the amount of phosphorylated src and FAK was monitored. For C3 exoenzyme, SMIFH2, CK666 and the activators, the actin organization was monitored by phalloidin-actin staining (Data not shown).

#### Mass Spectrometry

Cells were treated with cytochalasin D in serum free media followed by centrifugation; the collected pellet (membrane blebs) was subjected to lipid extraction using chloroform/methanol solvent system (Raghupathy et al., 2015). The lipid extract thus obtained was subjected to LC/MS by performing mass spectrometric analysis (Ramachandran et al., manuscript in preparation) on Q Exactive instrument (Thermo Fisher scientific). For quantitative measurements of the various phospholipid species in the 3 cell types, Please refer to Table S2.

#### Steady-state fluorescence emission anisotropy measurements and analysis

Homo-FRET based anisotropy measurements were carried out in TIRF mode (referred to as EA-TIRFM in the manuscript) on a NikonTE2000 or a Nikon TiE microscope with polarized laser excitation and fitted with an 100x 1.49 NA TIRF objective (or 100X 1.45 NA) with a dual camera imaging arrangement (Andor/Cairns Research) as described earlier (Ghosh et al., 2012). The emission was split into I*pa* (Parallel) and I*pe* (Perpendicular) components by means of a high-performance wire grid polarizer (Moxtek) and then collected onto two separate EMCCD cameras (Photometrics Evolve Delta or Cascade II).

Anisotropy is calculated using the formula

$$r=\frac{\left(\text{Ipa}-\text{Ipe}\right)}{\left(\text{Ipa}+2\text{Ipe}\right)}$$

Where I*pa* is the intensity measured in the parallel direction and I*pe* is the intensity in the perpendicular direction with respect to the plane of polarization of the excitation beam. For analysis, the images were background subtracted (1XM1-buffer image taken with identical imaging conditions) and the I*pa* and I*pe* images were aligned (Registration) using the affine transformation function in MATLAB (Mathworks, USA). An image of fluorescein in water was used to compute the G-factor (I*pa*/I*pe*) image, which was then multiplied pixel-to-pixel onto the I*pe* image. All high resolution anisotropy maps presented in the manuscript have been obtained by applying a threshold for the total intensity, and additionally smoothened by using a 2D averaging filter of size 3 (or 5) as indicated in the figure legends using custom written codes in MATLAB (Mathworks, USA). As such, the anisotropy LUT maps serve as a visual representation only and is not used for any quantification.

For the analysis, ~2x2 μm regions of interest (ROIs) were drawn on intensity flat regions of the membrane excluding endosomes using the ROI manager tool provided in Fiji (Schindelin et al., 2012), and the extracted intensities in the I*pa* and G-factor corrected I*pe* channels were used to compute the emission anisotropies for each ROI. The anisotropy values from several ROIs were binned based on total intensity values (I*pa*+2I*pe*) and a 2D plot the mean anisotropy in each intensity bin along with its standard deviation versus the intensity of each bin or scatter plots of mean and standard deviation overlaid with the individual ROI data points was plotted using GraphPad Prism 7 software (GraphPad).

For photobleaching experiments, YFP-GPI expressing CHO cells were subjected to time-lapse imaging using Multi-D acquisition in μ–Manager with an interval of 100-200ms between frames. Bleaching was done in widefield mode and TIRF images were acquired for quantification. For image analysis, the images were aligned using the first (un-bleached) frame and transformation was applied onto all subsequent frames. ~2X2 μm ROIs were randomly drawn from intensity flat regions from the membrane, and I*pa* and Gfactor corrected I*pe* intensities were recorded and the anisotropy of each ROI was computed as indicated above. The intensity of the ROI in the first frame was used to normalize the intensity in the other frames to get I/I0 ratios that are independent of starting intensity. This was used so that the data from multiple ROIs from multiple cells could be pooled together. A 2D plot of the binned I/I0 ratios versus mean anisotropy along with SD in each bin was then plotted.

For the quantification of whole cell anisotropy and area changes during cell spreading on FN, GFP-GPI expressing CHO cells were serum starved for 3 hours in the presence of 50 μg/ml cycloheximide and de-adhered and allowed to recover in suspension for 30 mins. ~100 cells were then added to the center of the FN coated coverslip dish that was pre-warmed and equilibrated on the 37°C temperature stage (Tokai Hit Co., Ltd) for at least 15 mins prior to imaging. As soon as a cell was located with bright field, time-lapse recording simultaneously in the two channels was started and the EA-TIRFM images were acquired with 15 min intervals. For the analysis, the images were aligned using the transformation matrix computed using the last frame of each time-series and the whole cell GFP-GPI anisotropy was calculated from the individual aligned I*pa* and Gfactor corrected I*pe* images and the area was quantitated from the total intensity image after intensity thresholding. The data from multiple cells were aligned with respect to the peak area change that is characteristic of the P1 phase of cell spreading. This was done to account for variability in the time at which the acquisition started and also variability in spreading dynamics between cells.

When imaging bright objects in TIRF, we typically find very faint residual fluorescence above our experimental background at one side of the object. This is usually observed along the direction of propagation of the evanescent field and arises due to the scattering by cellular features and resultant excitation of fluorescence outside the evanescent field (Fiolka, 2016). During post-processing (subtracting the experimental background) that signal is still present and therefore gets a pseudo-colored anisotropy value. However, these pixels are not included in the analysis because one can clearly distinguish the cell outline in the intensity image. For the image representation (In Figure 2A), we chose to include the anisotropy maps with a threshold for the background. Also, this results in a few pixels of high anisotropy toward the edge within the thresholded cell image. However, this does not contribute our estimations of change in anisotropy over time. Excluding pixels (0-20 pixels) from the edges of the segmented cell, does not change the whole cell anisotropy estimations (data not shown).

The absolute anisotropy values will differ when estimated for different fluorescent probes (that have different photo-physical properties) or when using microscope configurations that differ in the effective numerical aperture (NA). To document the contributions of these, we have performed the following set of experiments:

1.) To study the contribution of NA on the absolute anisotropy values, the same samples were imaged in microscope configurations with different effective NAs and the results are documented in Table S3. The effective NA was calculated from the point spread function (PSF) of sub-diffraction fluorescent beads (Molecular Probes/Invitrogen)

As is evident in Table S3, the absolute anisotropy values of the same sample in the different scopes are different. These differences in the values map closely to the effective NA in each case with the higher NA system exhibiting lower anisotropy values. Also, note that the dynamic range of the system (difference in values between control and mβ the absolute anisotropy values of the same sample in the different scopes are different (Ghosh et al., 2012).

2.) Effect of probe photo-physical properties (Table S3) on the absolute anisotropy values were estimated by comparing the anisotropy values obtained from different fluorescent probes reporting on the same experimental conditions and the day-to-day variations in absolute anisotropy value for the same probe was estimated by comparing the values obtained for the same probe under identical experimental conditions, but imaged on separate experimental dates.

Although the steady state anisotropy value varies across experiments, the relative change in the anisotropy values remains consistent (even with the usage of different probes). To account for such variation, we always include an internal control (cholesterol depleted cells on FN, cells on glass or Vin−/− cells; all of which have very few nanoclusters; Figures S2A and S2B; Figures 5A and 5B) in each experiment and instead of commenting on the measured absolute anisotropy values, we restrict our interpretations based on reporting the relative changes (reflecting a decrease (more nanoclustering) or increase (less nanoclustering) in anisotropy). Also note that although cells in suspension hardly have any GPI-AP nanoclusters (Figure 2A, 15 s panel), in order to have a sufficient cell spread area for quantification, the cells plated on glass were allowed to adhere and spread to a certain extent that resulted in the generation of some amount of GPI-AP nanoclusters (Figure 1C). Since this couldn’t be controlled, we observed a discrepancy in anisotropy value differences between glass and FN plated cells treated with mβCD across different experiments, that correlated with the extent of spreading observed on glass (For example compare differences in anisotropy values and cell spread area obtained on glass versus those obtained on FN+ mβCD in Figures 1B and 1C; Figures S2A and S2B). Also, note that with mβCD treatment on cells plated on FN, we were able to deplete only ~50% free membrane cholesterol (Figure S7F). This accounts for the low fraction of nanoclustering that was still observed under this condition (Figure 1C).

To monitor the extent of GPI-AP nanoclustering in cells plated on the cRGD functionalized SLBs prepared on chromium patterned surfaces (Figures 4H, 4I, and S4J), 1 μmX4 μm rectangular ROIs were drawn at the location of the pattern on the GPI-intensity image. The dip in the GPI-intensity along the lines served as a proxy marker for the localization of the chromium lines. For regions outside the pattern, 2 μmX 2 μm ROIs were drawn in the center of each pattern where the ligand is still mobile. These ROIs were then used to extract intensities from the I*pa* and Gfactor corrected I*pe* images and the anisotropy from each ROI was computed.

For the average line scan profiles presented in Figures 6F and S6I, ~1 μm line ROI were drawn perpendicular to the chromium pattern (in Figure 6F) or along the pattern (in Figure S6I) running through the center of vinculin clusters and the intensity profiles from the I*pa* and Gfactor corrected I*pe* images were extracted and GPI-anisotropy was computed. Since the vinculin clusters offer a good contrast, to combine multiple profiles, the vinculin pixel intensities along the line scan was first normalized to the maximum pixel intensity in each profile (to account for variability in expression/enrichment to clusters) and the vinculin intensity peaks were aligned and the mean GPI-intensity and anisotropy at various distances away from the peak were plotted by binning the data for the normalized vinculin intensity levels that fall sharply on either side of the peak.

For generating the average images presented in Figure 6E and 1 μmX1 μm ROIs were chosen such that each vinculin cluster was positioned at the center. These ROIs were used to crop the vinculin channel image and the GPI-AP I*pa* and Gfactor corrected I*pe* images. Since the vinculin clusters form on either side of the lines, the images were transformed so that the chromium pattern were always positioned along the left-hand side of each image. Also, since the vinculin intensities varied between clusters, the vinculin channel cropped images were normalized by dividing each pixel within the image to the maximum intensity value in each image. Using the raw transformed GPI-intensity images, the total intensity image and 3-pixel averaged anisotropy images were generated.

For estimating GPI-AP anisotropy within regions of the membrane that spatially correlated with vinculin clusters (Figures 6H and S6H), the vinculin clusters were first segmented using the blobSegmentThreshold.m function (Danuser laboratory, UT Southwestern Medical Center, USA) along with a gradient filter, and these masks were used to extract intensities from the GPI-AP I*pa* and Gfactor corrected I*pe* images using the region properties toolbox in MATLAB (Mathworks,USA). Manual regions drawn in the PM outside the segmented and the patterns were used to compute anisotropy outside clusters and patterns.

#### Fluorescence Correlation Spectroscopy (FCS) measurements and analysis

U2OS cells were transfected with GFP-Utr-AFBD (Gowrishankar et al., 2012) for 4-12 hours, de-adhered and plated onto FN coated dishes as described earlier, and placed on a point scanning confocal; Zeiss LSM 780 Confocal System. The temperature was maintained at 37°C with a temperature stage and objective heating collar (Tokai Hit,Co., Ltd). Cells with low to moderate expression levels were chosen for subsequent FCS experiments. Each cell measurement included at least six different, 10 s fluorescent traces that were obtained at the membrane attached to the substrate using a 40X 1.2 NA UV-VIS-IR C Achromat water-immersion objective. The back-focal plane of the objective was overfilled using 488nm laser in order to create a diffraction-limited confocal volume and was calibrated on each day before the experiment using the known diffusion coefficient of rhodamine 6G. The confocal spot was parked in the center of the field and each cell was moved to this position by moving the stage. The correct focal distance was determined each time as the z-focus where the initial estimate of counts per molecule was highest. Next, the emission photon stream was recorded with the same objective, de-scanned, through an aligned pinhole (32 μm), wavelength selected between 491-562 and detected on a gallium arsenide detector array.

Each 10 s trace (*I(t*)) was auto-correlated into an autocorrelation curve G(τ) using the Zeiss onboard auto-correlator which calculates the self-similarity through:

$$G\left(\tau \right)=\frac{\langle \delta I\left(t\right).\delta I\left(t+\delta \tau \right)\rangle }{{\langle I\left(t\right)\rangle }^{2}}$$

Here 〈〉 denotes the time-average, δ*I(t)* = *I(t)* - 〈*I(t)*〉 and τ is called the time-lag. Traces that showed effects of drift or sporadic high intense bursts were discarded from further analysis. As described earlier we find 3 diffusion timescales (Gowrishankar et al., 2012). The first timescale of about 20-120 μs corresponds to the triplet state, a second timescale (0.3-3ms) arising from free/unbound GFP-Utr-AFBD very close to the plasma membrane and a third timescale of around 10-200ms corresponding to GFP-Utr-AFBD bound to actin filaments diffusing through the confocal spot.

For all the data reported in this manuscript we fitted each (about 70) raw 10 s autocorrelation (G(τ)) versus time-lag (τ 5·10^−6^-2 s) to:

$$G\left(\tau \right)=\frac{1}{N}.G_{T}\left(\tau \right).G_{D}\left(\tau \right)$$

N reflects the number of moving particles in the confocal volume and G_T_(τ) is correlation function associated to blinking/triplet kinetics:

$$G_{T}\left(\tau \right)=\left(\frac{1+T}{1-T}\right).e^{\left(-\frac{\tau }{\tau T}\right)}$$

Where T is the fraction of molecules in the dark state and τt corresponds to the lifetime of the dark state. G_D_(τ) is correlation function associated to diffusion which in this case contains two diffusional timescales:

$$G_{D}\left(\tau \right)=f.G_{D1}\left(\tau \right)+\left(1-f\right).G_{D2}\left(\tau \right)=f.{\left(1+\frac{\tau }{\tau_{D1}}\right)}^{-1}.{\left(1+\frac{\tau }{S^{2}.\tau_{D1}}\right)}^{-1/2}+\left(1-f\right).{\left(1+\frac{\tau }{\tau_{D2}}\right)}^{-1}$$

The fraction f corresponds to the intracellular diffusing pool of unbound GFP-Utr-AFBD that has a timescale of t_D1_. S is the structure factor that accounts for timescales arising from the fact that the intracellular GFP-Utr-AFBD diffuses in a volume rather that a plane. Free fitting this parameter converges the value to about 0.2, consistent with earlier reports. In order to constrain the number of free parameters we decided to fix this value to 0.2. The diffusion time associated with diffusing GFP-Utr-AFBD that is bound to actin filaments is finally calculated with τ_D2_.

#### Laurdan probe based membrane ordering measurements and analysis

For the measurement of membrane order in living cells, we used the previously characterized solvatochromic probe Laurdan (6-lauryl-2-dimethylamino naphthalene), that undergoes a 50 nm red-shift in its emission maxima in the polar environment of liquid disordered phases (Owen et al., 2011).This shift in the emission profiles between ordered and disordered phases can be quantified as a ratiometric estimate based on fluorescence intensities collected in these two spectral channels referred to as generalized polarization (GP). Laurdan imaging was implemented on a Yokogawa CSU-22 spinning disc unit confocal microscope. 10mM Laurdan stocks (Sigma Aldrich) was prepared in anhydrous DMSO and stored in airtight amber colored vials in a desiccator. CHO cells (WT, PGAP2&3 mutant or rescue) was grown in coverslip bottom dishes and labeled with 40μM Laurdan in 1XM1-1mg/ml glucose for 5 mins at 37°C. The quick labeling procedure reduces the buildup of fluorescence signal in internal pools and reduces sticking of the probe to the dish. The cells were washed several times in 1XM1-glucose to reduce background non-specific staining. The samples were excited with 405 nm laser line and 100X high resolution images were recorded simultaneously by splitting the emission using a 458nm long pass dichroic (ZT458rdc;Chroma Technology Corp) in two separate spectral channels Ch1 (435/40nm) (FF02-435/40-25;Semrcok) and Ch2 (504/37nm) (FF01-504/37-25; Semrock) imaged onto two separate EMCCD Cameras (AndorIxon + 897 EMCCD). For background, we imaged a dish containing only 1XM1-glucose buffer under identical imaging conditions. 1 μM Laurdan in DMSO was imaged to estimate the Gfactor (Owen et al., 2011) of the system.

It is estimated using the formula:

$$\text{Gfactor}=\frac{{\text{GP}}_{\text{ref}}+{\text{GP}}_{\text{ref}}.{\text{GP}}_{\text{mes}}-{\text{GP}}_{\text{mes}}-1}{{\text{GP}}_{\text{mes}}+{\text{GP}}_{\text{ref}}.{\text{GP}}_{\text{mes}}-{\text{GP}}_{\text{ref}}-1}$$

Where GP_mes_ is the experimentally determined GP value of Laurdan in DMSO and GP_ref_ is a reference value for Laurdan GP which is conventionally fixed at 0.207 (Gaus et al., 2006).

The GP_mes_ was determined every experiment and the computed Gfactor value was uniformly applied to the Ch2 images.

The GP was then calculated using the formula:

$$\text{GP}=\frac{I_{\text{Ch}1}-G.I_{\text{Ch}2}}{I_{\text{Ch}1}+G.I_{\text{Ch}2}}$$

Laurdan GP values were calculated for each pixel of the image to obtain the high resolution Laurdan GP spatial maps of membrane ordering presented in this manuscript. ROI analysis for quantification was performed by drawing ~2X2 μm ROIs and extracting intensities in the two channels to compute the GP value offline.

#### Cell spreading assay and Adhesion size estimation

Tissue culture dishes were cleaned by treating with 1% Hellmanex III (Hellma Analytics) for 30 mins at 37°C and then thoroughly washing with sterile MilliQ water followed by UV sterilization. The dishes were then coated with 10 μg/ml human plasma fibronectin (Merck Millipore) at 37°C for 1 hour and blocked with 1% w/v BSA for additional 30 mins and subsequently washed thrice with 1XPBS and once with phenol free incomplete media. The cells were de-adhered with TrypLE reagent (Invitrogen), pelleted down and re-suspended in appropriate amount of incomplete media and left at 37°C for 30 mins for recovery and subsequently plated onto the prepared fibronectin-coated dishes. After 5 mins of initial cell adherence, the unbound cells were washed off and the dishes were transferred back to 37°C/5% CO_2_ incubator. The cells were imaged in phase contrast with a 20X objective at each of the indicated time points. For experiments involving the cells expressing various Vinculin-GFP constructs, the transfected Vin ^−/−^ cells were and allowed to spread onto FN coated dishes and either fixed with 4% paraformaldehyde (PFA) immediately (0 hr) or fixed after 35 mins of incubation at 37°C/5% CO_2_. The transfected cells were imaged using the 20X objective in 488 (GFP) channel along with corresponding phase contrast images. For quantification of cell spread area, the cells were marked manually and the mean cell area was extracted using the ROI manager tool in Fiji (Schindelin et al., 2012).

For adhesion size estimation, cells were allowed to spread on FN-coated glass coverslips for 90 mins and subsequently fixed with 2.5% PFA for 20 mins at RT. Cells were then permeabilized with 0.1% Triton X-100 (Sigma Aldrich) for 10 mins at RT and then blocked with 1% w/v BSA. The FAs were labeled by incubation with mouse anti-paxillin antibody (BD Transduction Laboratories) and subsequently probed with a fluorescently tagged anti-mouse antibody (Jackson ImmunoReserach Labs., Inc) and the F-actin was labeled with Phalloidin-Alexa 568 (Thermo Fisher Scientific). TIRF images were acquired with a 100X 1.45 NA objective. To estimate adhesion sizes, background (1XPBS) subtracted paxillin images were used to segment adhesions using the blogSegmentThreshold MATLAB code (Danuser laboratory, UT Southwestern Medical Centre, USA) along with a gradient filter. The area of each adhesion was extracted using the region properties tool available in MATLAB (Mathworks, USA).

### Quantification and Statistical Analysis

Unless explicitly stated, each reported experimental condition includes data collected from at least two technical replicates (2 independent dishes for each condition). For data quantification, ROI (~100 per condition) based analysis was done on 10-30 cells across the technical replicates. Additionally, each experiment reported here was performed at least twice (biological replicates), but due to variability in the absolute anisotropy values across experimental dates (See Table S3), only the dataset from one experiment (along with appropriate internal controls) is presented here.

In Table S4, the sample sizes used for all the plots and statistical analysis are shown. Please note that in sample size estimations, each ‘field’ represents a 512 X 512 pixel (~50X50 μm with 100X magnification) image obtained from an EMCCD camera that contains at least one cell per field. Unless otherwise stated, ‘ROIs’ represent a 20 X 20 pixel (~2X2 μm) region of interest drawn over the indicated number of fields that were pooled from technical duplicates of each condition. ‘Cells’ denote the number of cell images used in the analysis.

Since we were interested in performing statistical analysis across multiple perturbations, we performed Kruskal-Wallis statistical test with the level of significance (α) set to 0.01 using GraphPad Prism 7 software (GraphPad). The Kruskal-Wallis test is a non-parametric test used to compare the mean rank of each column with the mean rank of every other column. Dunn’s correction is applied for multiple comparison and the multiplicity corrected p values is shown the Table S4 along with the significance and exact p values. In cases, where we are comparing only across two experimental conditions, we compare the ranks using the non-parametric Mann-Whitney two-tailed p value test with a confidence level of 99%. In this case, the p value is also indicated in the figure panel itself. n.s p > 0.05, *p ≤ 0.05, **p ≤ 0.01, ***p ≤ 0.001, ****p ≤ 0.0001.

## Supplementary Material

Supplemental Information can be found online at https://doi.org/10.1016/j.cell.2019.04.037.

Table S4

Video S1

Video S2

Table S2

Video S3

Table S1

Table S3

Supplemental Figures

## Highlights

- Integrin signaling triggers RhoA-Formin to drive acto-myosin based nanoclustering
- Force-mediated vinculin activation promotes GPI-anchored protein nanoclustering
- GPI-anchored protein nanoclusters regulate Fibronectin-mediated integrin function
- Integrins represent an active element of the actin-membrane composite model
