# Supplementary material for: Integrin Mechano-chemical Signaling Generates Plasma Membrane Nanodomains that Promote Cell Spreading
Source: Cell. Author manuscript; Available in PMC 2019 Nov 26. (PMC6879320; doi:10.1016/j.cell.2019.04.037)
Supplement: Table S2 [file EMS84939-supplement-Table_S2.pdf]

Table S2: Quantitative measurements (pmoles/µg of protein) of phospholipid species in WT, PGAP2/3 mutant and Rescue CHO cells; Related to STAR Methods, Mass Spectrometry

Lipid Species		WT	PGAP2/3 mutant	Rescue
**PI(27:3e)**	PI	0.125563	0.113835	0.080251
**PI(38:6)**	PI	0.868918	0.867472	0.821913
**PI(19:2)**	PI	0.853849	0.808515	0.849968
**sPI(24:6/14:1)**	PI	1.220225	1.291897	1.210407
**PI(18:1/20:4)**	PI	0.532498	0.515193	0.530848
**PI(38:5)**	PI	0.555556	0.511464	0.546388
**PI(38:3)**	PI	0.097476	0.093842	0.090076
**PI(34:6p)**	PI	0.38199	0.333103	0.369383
**PI(33:1p)**	PI	0.432825	0.400921	0.430856

Lipid Species		WT	PGAP2/3 mutant	Rescue
**PS(27:0p)**	PS	0.601937	0.599336	0.599991
**PS(29:2e)**	PS	0.615304	0.613274	0.61196
**PS(36:3p)**	PS	0.582513	0.57958	0.581684
**PS(44:10)**	PS	0.184758	0.118314	0.12227
**PS(18:1/18:1)**	PS	0.140208	0.113078	0.097644
**PS(40:5)**	PS	2.058949	2.051079	2.051539
**PS(17:0/17:0)**	PS	0.580006	0.578871	0.576012
**PS(40:4)**	PS	0.416677	0.311655	0.330858
**PS(18:0/18:1)**	PS	4.210633	4.188382	4.207938
**PS(38:1p)**	PS	0.357987	0.323507	0.356884
**PS(38:2e)**	PS	0.296373	0.291156	0.212621
**PS(40:2)**	PS	0.639532	0.614241	0.611264
**PS(40:0)**	PS	0.118904	0.14816	0.111733
**PS(42:2)**	PS	0.534892	0.534627	0.53391
**PS(34:1/8:0)**	PS	0.998981	0.991286	0.989176

Lipid Species		WT	PGAP2/3 mutant	Rescue
**PE(16:2/22:5)**	PE	0.178700488	0.178242651	0.178100763
**PE(18:1p/20:4)**	PE	1.380253659	1.379484726	1.377938015
**PE(18:2p/20:5)**	PE	0.197303415	0.196874083	0.197296366
**PE(24:5/18:2)**	PE	0.101756098	0.101731182	0.101536122
**PE(36:2p)**	PE	0.189955122	0.189688761	0.18952284
**PE(36:4)**	PE	0.194025366	0.193664707	0.193540519
**PE(37:3)**	PE	1.166552195	1.165643804	1.166293924

**PE(37:4)**	PE	1.158821463	1.15767147	1.158744183
**PE(37:4)**	PE	1.841365854	1.835346782	1.84089771
**PE(38:4)**	PE	0.169685854	0.169330259	0.169241221
**PE(40:4)**	PE	1.129045854	1.128251566	1.128847664
**PE(40:7)**	PE	1.105771707	1.104953199	1.105378321
**PE(41:4)**	PE	1.244019512	1.241306436	1.243161527
**PE(41:6)**	PE	1.332995122	1.331279539	1.332598107
**PE(43:2)**	PE	1.159067317	1.157974063	1.158448366
**PE(43:4)**	PE	1.572839024	1.572372719	1.571396641
**PE(45:6)**	PE	0.166134634	0.166121422	0.166036183
**PE(45:8)**	PE	0.4008	0.400312085	0.400266076
**PE(8:0e/10:0)**	PE	0.16313	0.151858	0.134369

**Lipid Species**		**WT**	**PGAP2/3 mutant**	**Rescue**
**PC(10:0/21:1)**	PC	0.395368	0.31066	0.377856
**PC(13:1/21:2)**	PC	0.507177	0.417399	0.375112
**PC(16:1/18:1)**	PC	7.838852	7.780404	7.809166
**PC(18:2/20:4)**	PC	1.190752	1.165202	1.185957
**PC(18:4/22:2)**	PC	0.344968	0.33304	0.272525
**PC(18:4/22:3)**	PC	0.341788	0.450498	0.472123
**PC(19:1/18:1)**	PC	0.753792	0.705917	0.584523
**PC(20:1/18:3)**	PC	1.093133	1.03925	0.78479
**PC(24:3/16:2)**	PC	0.40124	0.456649	0.487511
**PC(32:2)**	PC	0.317811	0.277124	0.248439
**PC(34:2e)**	PC	0.775811	0.724663	0.599911
**PC(35:2)**	PC	1.186103	1.176977	1.185716
**PC(36:2)**	PC	12.82743	12.72759	12.79822
**PC(36:3)**	PC	2.862502	2.85413	2.854215
**PC(36:4)**	PC	3.077801	3.059988	2.803568
**PC(36:4)**	PC	3.410537	3.396013	3.397703
**PC(38:2)**	PC	2.038738	2.037047	2.033363
**PC(38:3)**	PC	0.727858	0.684827	0.61463
**PC(38:4)**	PC	0.614337	0.59256	0.483497
**PC(38:5)**	PC	2.084733	2.082645	2.069492
**PC(38:6e)**	PC	0.701191	0.676626	0.684389
**PC(38:7)**	PC	0.31145	0.354716	0.299955
**PC(40:6p)**	PC	0.247594	0.220826	0.158698
**PC(8:0/10:0)**	PC	0.526505	0.426479	0.448483
