# Supplementary material for: Integrin Mechano-chemical Signaling Generates Plasma Membrane Nanodomains that Promote Cell Spreading
Source: Cell. Author manuscript; Available in PMC 2019 Nov 26. (PMC6879320; doi:10.1016/j.cell.2019.04.037)
Supplement: Table S1 [file EMS84939-supplement-Table_S1.pdf]

Cell, Volume *177*

Supplemental Information

Integrin Mechano-chemical Signaling Generates Plasma Membrane Nanodomains that Promote Cell Spreading

Joseph Mathew Kalappurakkal, Anupama Ambika Anilkumar, Chandrima Patra, Thomas S. van Zanten, Michael P. Sheetz, and Satyajit Mayor

Table S1: Mobile fractions of cRGD conjugated Neutravidin DyLight 650 on various surfaces; Related to STAR Methods, Preparation of cRGD functionalized Supported Lipid Bilayers (SLBs).

**cRGD bound to Neutravidin DyLight 650**	**Mobile Fraction Mean ± SD (N=2)**
**Continuous SLBs**	0.902 ± 0.02
**Nanopatterened SLBs**	0.148 ± 0.019
**PLL-g-PEG coated Glass**	0.091 ± 0.006
