# Supplementary material for: Integrin Mechano-chemical Signaling Generates Plasma Membrane Nanodomains that Promote Cell Spreading
Source: Cell. Author manuscript; Available in PMC 2019 Nov 26. (PMC6879320; doi:10.1016/j.cell.2019.04.037)
Supplement: Table S3 [file EMS84939-supplement-Table_S3.pdf]

Table S3: Characterization of causes for the variability in EA-TIRFM anisotropy values; Related to STAR Methods; Steady-state fluorescence emission anisotropy measurements and analysis

Effect of Effective Numerical Aperture (NA) on emission anisotropy:

Microscope cofiguration	Effective NA	Anisotropy value on FN	Anisotropy value after mβCD treatment	Relative Aniso change	Calculated FRET efficiency (Fraction)
**100X/TIRF**	1.1	0.1739 (0.005)	0.1896 (0.005)	0.0157	0.25
**100X/TIRF**	0.85	0.1917 (0.005)	0.218 (0.004)	0.0263	0.37
**100X/Confocal**	0.73	0.1926 (0.006)	0.226 (0.01)	0.034	0.37

Effect of probe photophysical properties on emission anisotropy:

Probe used	Mean anisotropy value in Vin-/- cells (SD)	Mean anisotropy value in Vinculin add back cells (SD)	Relative anisotropy change
**GFP-GPI (Figure 5B)**	0.146 (0.006)	0.098 (0.003)	0.048
**GFP-GPI (Figure S6B)**	0.165 (0.006)	0.099 (0.005)	0.066
**Alexa-568-FLAER (Figure 5F)**	0.115 (0.003)	0.065 (0.003)	0.050
**mRuby2-GPI (Figure 6D)**	0.192 (0.0092)	0.157 (0.0032)	0.035
**mRuby2-GPI (Figure S5E)**	0.185 (0.0045)	0.138 (0.0063)	0.047

Probe used	Mean anisotropy value of cells on FN (SD)	Mean anisotropy value of cells on FN after mβCD treatment (SD)	Relative anisotropy change
**GFP-GPI (Figure1D)**	0.159 (0.002)	0.182 (0.004)	0.0233
**YFP-GPI (Figure 1F)**	0.174 (0.005)	0.189 (0.005)	0.0157
