# Supplementary material for: Integrin Mechano-chemical Signaling Generates Plasma Membrane Nanodomains that Promote Cell Spreading
Source: Cell. Author manuscript; Available in PMC 2019 Nov 26. (PMC6879320; doi:10.1016/j.cell.2019.04.037)
Supplement: Supplemental Figures [file EMS84939-supplement-Supplemental_Figures.pdf]

Supplemental Figures

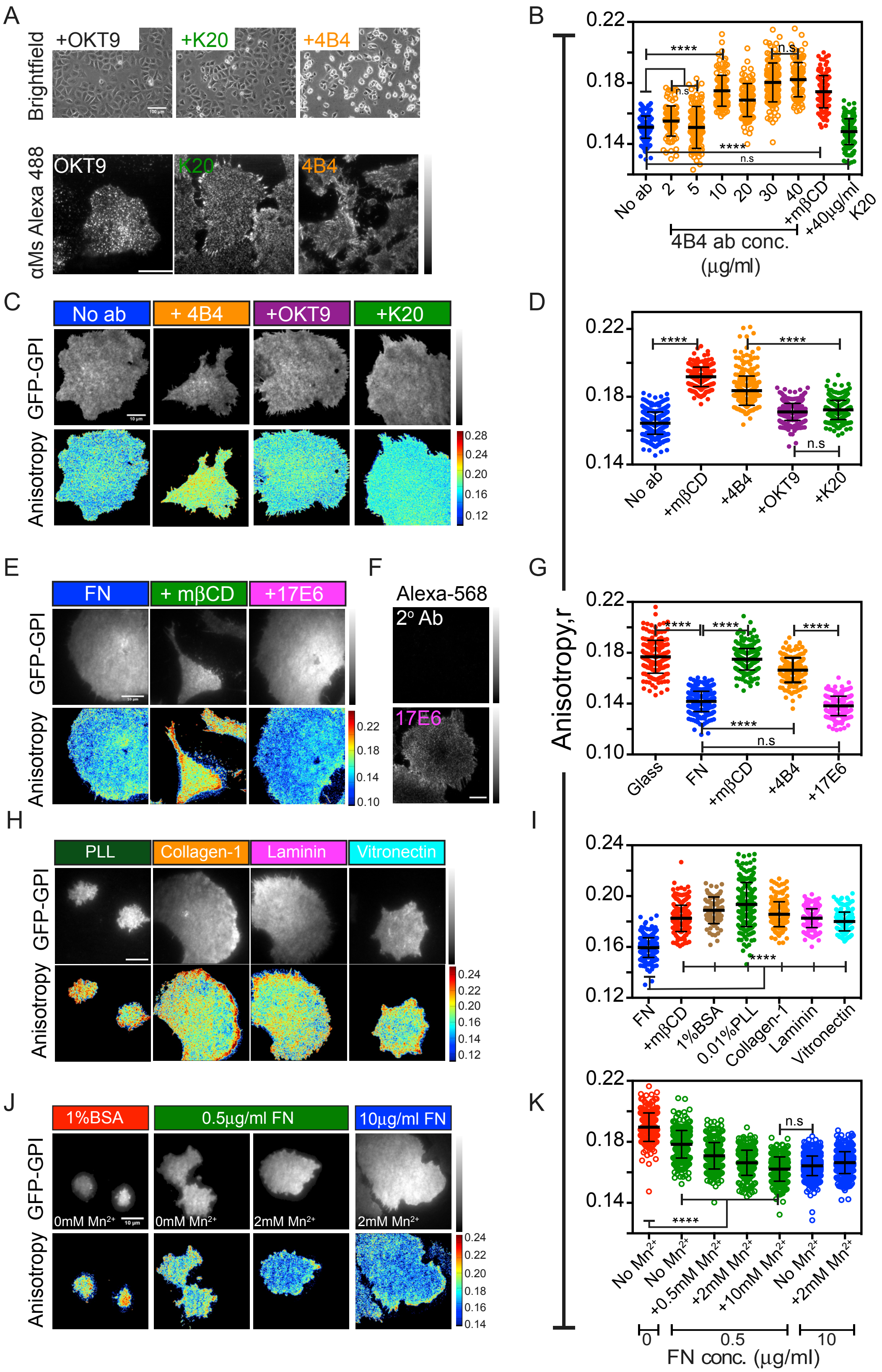

(legend on next page)

Figure S1. Increase in GPI-AP Nanoclustering on FN Occurs in a β1 Integrin-Dependent Manner and Requires Activated Integrin, Related to Figure 1

(A-B) Brightfield images. Top panel: Pre-treatment of U2OS cells with the β1 integrin function blocking antibody (4B4) results in defective cell spreading response on FN. In contrast, pre-treatment of U2OS cells with a non-function perturbing β1 integrin antibody (K20) or an antibody targeting a non-related receptor TfR (OKT9) has no effect on cell spreading. Fluorescent images. Bottom panel: While the pre-treated K20 and 4B4 integrin antibodies localize to focal adhesions (FAs) that are formed post-cell spreading, anti-TfR antibody (OKT9) localized to punctate structures distributed throughout the cell surface when imaged in TIRF. Scale bar 100 μm (top panel) and 10 μm (bottom panel). (B) Scatter dot plot with mean of ROI anisotropy values obtained from GFP-GPI expressing U2OS cells (U2OS GG) plated on FN either without (blue), or with treatment with 10mM mβCD (red), or with pre-treatment while in suspension with increasing amounts of indicated antibodies before plating in their continued presence; 4B4 (2-40 μg/ml; orange open circles), K20 (40 μg/ml; green). Scale bar 10 μm. (C-D) Representative intensity and steady state anisotropy images (C) and scatter dot plot with mean of ROI anisotropy values (D) obtained from U2OS GG cells plated on FN without (blue) or with treatment with 10mM mβCD (red), or antibodies, 4B4 (orange), OKT9 (purple) or K20 (green), and plated on FN in the continued presence of the antibodies. Scale bar 10 μm. (E-G) Representative intensity and steady state anisotropy images (E), and scatter dot plot with mean of ROI anisotropy values (G) obtained from U2OS GG plated on glass (red) or on FN before (blue) or after subsequent treatment with 10mM mβCD (green), with pre-treatment while in suspension with 10 μg/ml of function blocking antibody directed against either the β1 integrin (4B4;orange) or the αV integrin (17E6;magenta) and plated on FN in the continued presence of the antibody. (F) TIRF intensity images from U20S GG cells, pre-treated without (top) or with 17E6 antibody (bottom), and subsequently plated on FN and fixed, stained with Alexa568-labeled 2°antibodies. Scale bars, 10 μm. Note the robust staining of the cell surface with 17E6 antibody. (H-I) Representative intensity and steady state anisotropy images (H) and scatter dot plot with mean of ROI anisotropy values (I) obtained from U2OS GG cells plated on glass coverslips coated either with Poly-L-lysine (0.01% PLL; green) or rat tail Collagen-1 (50 μg/ml; orange), EHS Laminin (20 μg/ml, magenta), Vitronectin (10 μg/ml; cyan) or on FN (10 μg/ml; blue, red) before (blue) or after treatment with 10mM mβCD (red). Scale bar 10 μm. (J-K) Representative intensity and steady state anisotropy images (J) and scatter dot plot with mean of ROI anisotropy values (K) obtained from U2OS GG cells plated on glass blocked with 1%BSA without Mn^{2+} (red) or on 0.5 μg/ml FN without (green) or with 0.5mM or 2mM Mn^{2+} or on 10 μg/ml FN (blue) with or without 2mM Mn^{2+} as indicated. Scale bar 10 μm. All error bars represent SD. n.s $p > 0.05$, *$p \leq 0.05$, **$p \leq 0.01$, ***$p \leq 0.001$, ****$p \leq 0.0001$. Sample size and p values are provided in Table S4.

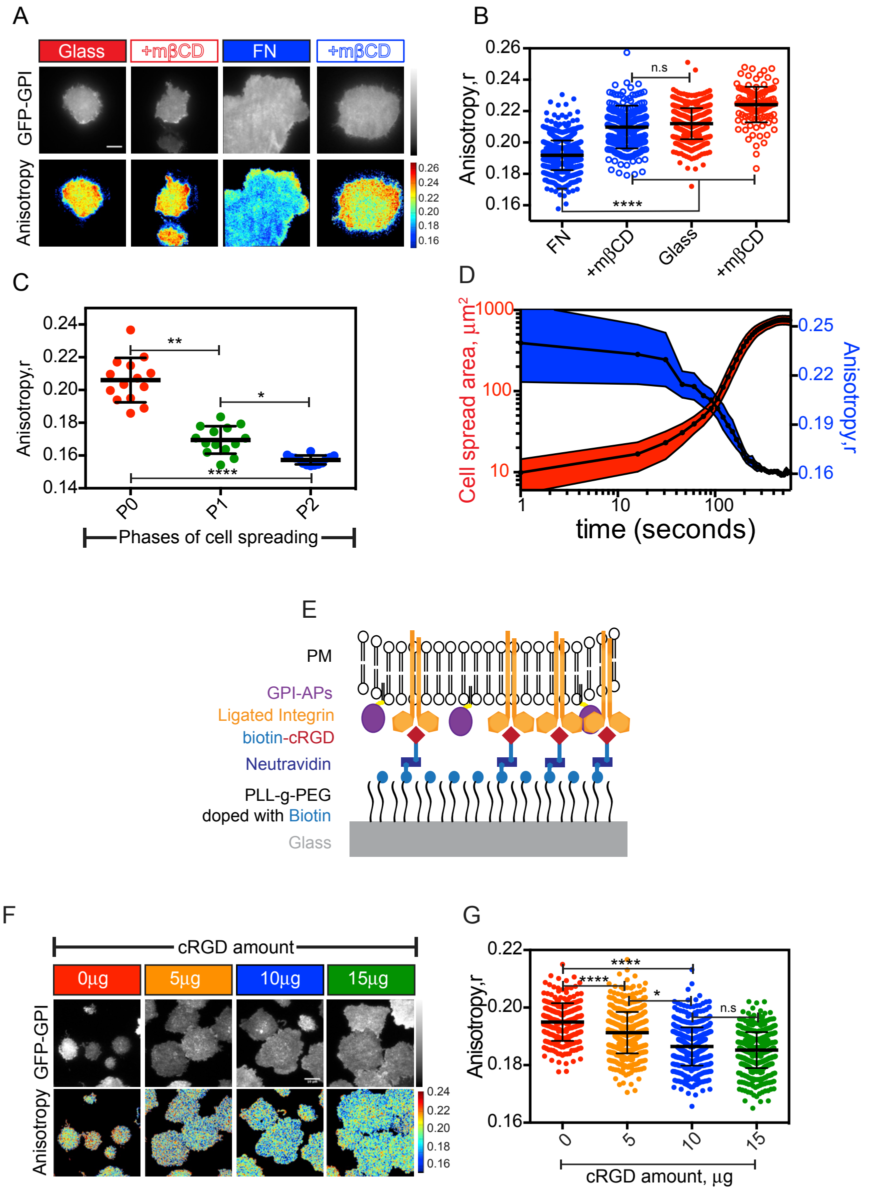

Figure S2. Integrin-FN or cRGD Engagement Leads to Enhanced GPI-AP Nanoclustering, Related to Figure 2

(A-B) Representative intensity and steady state anisotropy images (A) and scatter dot plot with mean of ROI anisotropy values (B) obtained from GFP-GPI expressing CHO cells plated on glass (red) or FN-coated glass (blue) without (closed circles) or with subsequent treatment with 10mM mβCD (open circles). Note that the cells plated on glass and those on FN treated with mβCD have the same steady-state anisotropy value and therefore either of these conditions may be used as an internal control for a condition where nanoclusters are highly reduced. (C, D) Scatter dot plot (C) with the mean anisotropy values during the indicated phases of cell spreading quantified by drawing line ROIs on the intensity kymographs and extracting the corresponding values from the anisotropy kymographs. (D) Plot of whole cell spread area (red curve) with corresponding raw anisotropy values (blue curve) as a function of spreading time (log time-x axis) of GFP-GPI expressing CHO cells (n = 4) on FN. The black curve represents the mean and the shaded area represents SEM. (E-G) Schematic (E) of cRGD functionalized PLL-g-PEG-biotin coated glass surfaces. Neutravidin acts as a sandwich layer between PLL-g-PEG-biotin and cRGD-biotin. Cell expressing GFP-GPI and appropriate integrins are added onto this surface. Additional details can be found in the STAR Methods. (F-G) Representative intensity and anisotropy images (F) and scatter dot plots with mean of ROI anisotropy values (G) obtained from GFP-GPI expressing CHO cells plated for 60 mins on PLL-g-PEG-biotin with 15 µg unlabeled neutravidin alone (red), or additionally functionalized with either 5 µg (orange), 10 µg (blue) or 15 µg (green) of cRGD-biotin prior to addition of cells. Scale bar 10 µm in all panels. All error bars represent SD. n.s $p > 0.05$, *$p \leq 0.05$, **$p \leq 0.01$, ***$p \leq 0.001$, ****$p \leq 0.0001$. Sample size and p values are provided in Table S4.

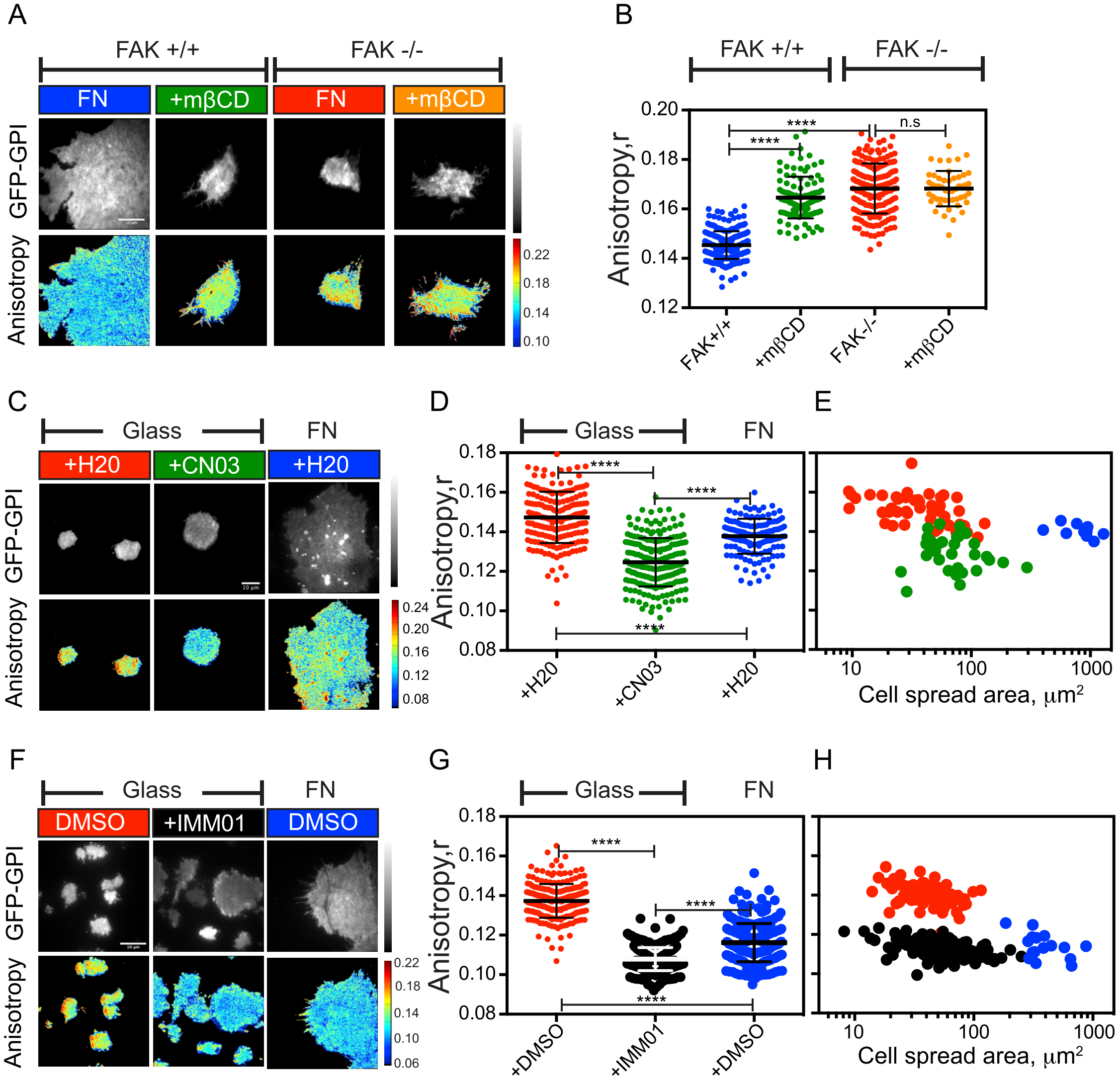

Figure S3. Effect of Perturbations of Downstream Targets of Integrin Signaling on GPI-AP Nanoclustering, Related to Figure 3

(A-B) Representative intensity and steady-state anisotropy images (A) and scatter dot plot (B) with mean of ROI anisotropy values obtained from GFP-GPI expressing FAK+/+ (blue, green) or FAK−/− (red, orange) mouse embryonic fibroblasts (MEFs) cells plated on 10 μg/ml FN without (blue, red) or with treatment with 10mM mβCD (green, orange). (C-E) Representative intensity and steady state anisotropy images (C), scatter dot plot with mean ROI anisotropy values (D) and 2-D scatterplot of whole cell spread area versus anisotropy (E) obtained from GFP-GPI expressing CHO cells pretreated with vehicle (+H2O) alone and plated either on FN (blue) or on glass (red) or with 10 μg/ml RhoA activator (+CN03) and plated on glass (green). (F-H) Representative intensity and steady state anisotropy images (F), scatter dot plot with mean ROI anisotropy values (G) and 2-D scatterplot of whole cell spread area versus anisotropy (H) obtained from GFP-GPI expressing CHO cells pretreated with the vehicle DMSO alone and plated either on FN (blue) or on glass (red) or with 10 μM formin agonist (+IMM01; black) and plated on glass. Note that anisotropy values were uncorrelated with cell spread area on glass with and without the activators in both the cases. Scale bars, 10 μm in all panels. All error bars indicate SD. n.s $p > 0.05$, *$p \leq 0.05$, **$p \leq 0.01$, ***$p \leq 0.001$, ****$p \leq 0.0001$. Sample size and p values are provided in Table S4.

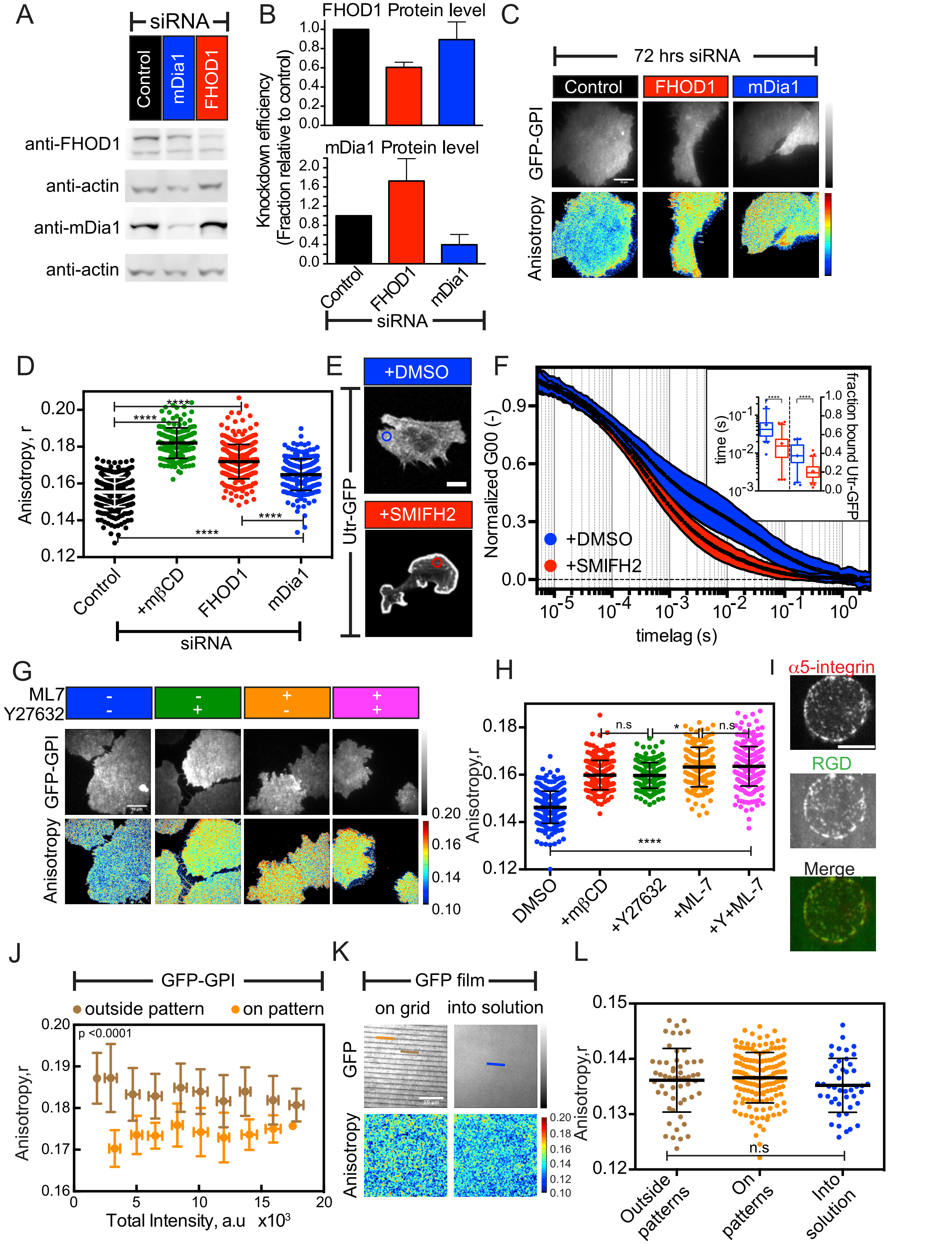

(legend on next page)

Figure S4. Integrin Activation Alters Cortical Acto-myosin Activity, Related to Figure 4

(A and B) Immuno-blot (A) of the indicated proteins after siRNA mediated knockdown in U2OS GG cells transfected for 72 hours with 5 nmoles SMARTpool of either control scrambled siRNA (black) or siRNA against the formins FHOD1 (red) or mDia1 (DIAPH1; blue) and probed with the indicated antibodies. (B) Quantification of the blots (from two independent RNAi experiments in each case) indicate a knockdown of $\sim$40% FHOD1 protein levels and $\sim$60% in the case of mDia and a compensatory $\sim$60% increase in expression of mDia1 when FHOD1 was knocked down. The data is normalized first to the corresponding intensities of β-actin in each well (loading control) and then to the levels of the protein in the control siRNA condition. The error bars represent SD (C,D) Representative intensity and steady-state anisotropy images (C) and scatter dot plot (D) with mean of ROI anisotropy values obtained from U2OS GG cells plated on 10 μg/ml FN after treatment for 72 hours with 75 ngs of either control siRNA (black, green) or FHOD1 siRNA (red) or mDia1 siRNA (blue), and subsequently treated with 10mM mβCD (green) and imaged. Scale bar 10 μm. (E-F) Confocal images (E) and autocorrelation decays (F; mean, black curve; SD, shaded area) of GFP-tagged Utrophin actin filament binding domain (GFP-Utr AFBD) in cells plated on FN (blue) or pre-treated and plated on FN in the presence of 10 μM formin inhibitor SMIFH2 (red). FCS data was collected from regions in the cell periphery devoid of stable actin filaments as indicated (blue or red circles in E). Scale bar 10 μm. Note the loss of the slow diffusion timescales ($\sim$10ms) in cells treated with the formin inhibitor. Inset in F shows the distribution of timescale and fraction of the slow moving population associated with moving actin filaments. (G-H) Representative intensity and steady-state images (G) and scatter dot plot with mean of ROI anisotropy values (H) obtained from CHO cells stably expressing GFP-GPI plated on 10 μg/ml FN after treatment with DMSO (control; blue) or 10mM mβCD (red) or pre-treated with 20 μM of ROCK inhibitor Y-27632 (green), 20 μM of MLCK inhibitor ML7 (orange) or both ROCK and MLCK inhibitors (20 μM each; +Y+ML-7;magenta) and subsequently plated on FN in the presence of the drugs. Scale bar 10 μm. (I) CHO-B2 cells stably expressing GFP-α5(β1) integrin plated on continuous SLBs functionalized with mobile cRGD. The cRGD bound neutravidin (labeled with DyLight 650) serves as a proxy marker for the integrin clusters since they co-localize with the GFP-α5 integrin clusters created on the SLBs (bottom panel; merge). Scale bar 5 μm. (J) Plot of mean anisotropy values at various binned total intensities obtained from GFP-GPI expressing CHO cells plated on cRGD functionalised SLBs formed on nanopatterned surfaces. 20x20 μm ROIs were drawn at the center of the grid pattern where the cRGD ligand is mobile (orange) or 1 μm X 4 μm ROIs (orange) were drawn on the membrane above the grid patterns. Note the anisotropy values are independent of the fluorescence intensity despite the decrease in GFP-GPI intensity observed on the membrane above the patterns (See Figure 4H). This decrease is due to opaque nature of the chromium lines. (K-L) Representative intensity and anisotropy images (K) and scatter dot plot with mean of ROI anisotropy values (L) obtained from a film of GFP-solution on chromium nanopatterned glass surfaces taken at the grid focus and quantified on the patterns (orange) or from areas outside the patterns (brown) focused into the solution and randomly sampled (blue). Scale bar 10 μm. Note that there is a decrease in GFP-intensity at the grids lines in focus, and this does not contribute to any difference in anisotropy over the whole image; the anisotropy values deep in solution on over the grids is identical. This confirms that the observed decrease in GFP-GPI anisotropy was genuine, and not a consequence of changes in GFP intensity. All error bars indicate SD. n.s $p > 0.05$, *$p \leq 0.05$, **$p \leq 0.01$, ***$p \leq 0.001$, ****$p \leq 0.0001$. Sample size and p values are provided in Table S4.

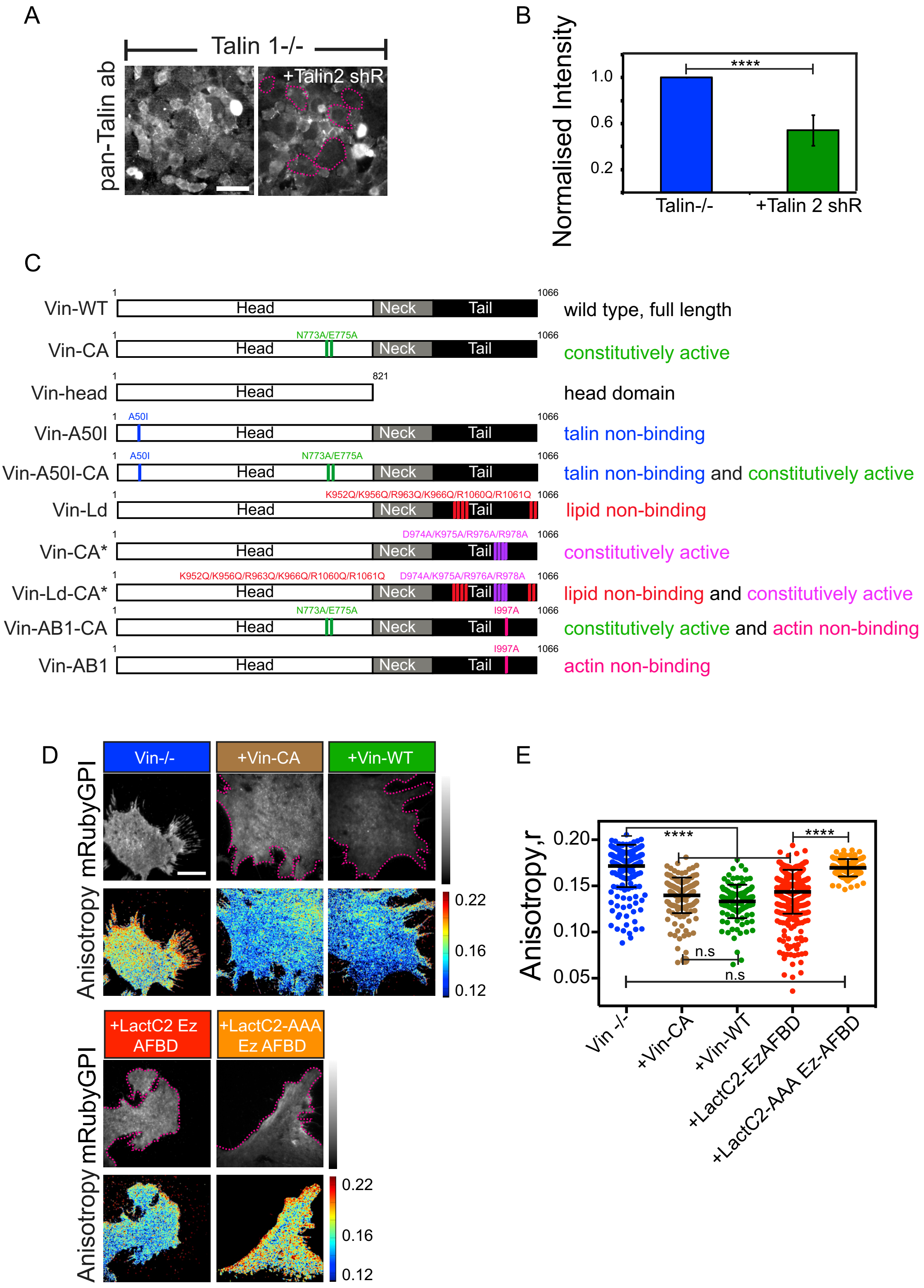

Figure S5. Characterization of talin1$^{-/-}$ MEFs, Schematic of Vinculin Mutant Constructs Used in This Study, and Restoration of GPI-AP Nanoclustering in Vin$^{-/-}$ Cells by Introduction of Artificial Linker, Related to Figure 5

(A-B) Representative intensity images (A) and corresponding bar graph (B) quantifying the mean endogenous talin levels using a pan-talin antibody in talin1−/− cell line in the presence (green), or absence (blue) of GFP-tagged talin2 shRNA after normalization to the levels in the talin1−/− control. The magenta dotted lines

(legend continued on next page)

mark the GFP cells expressing the plasmid encoding the shRNA. The data shows a ~45% decrease in total talin levels in talin −/− cells subsequently treated with talin2 shRNA. Scale bar 100 μm. (C) Schematic depicting the various vinculin variants used in this study, and the typical characteristics of each molecule. (D,E) Representative intensity and steady-state anisotropy images (D) and scatter dot plot with mean of ROI anisotropy values (E) obtained from mRuby-2 expressing (12-14 hours) Vin −/− cells plated on FN (Blue) or co-transfected for the same time with either Vin-WT (green) or Vin-CA (brown) or LactC2-Ez AFBD (red) or LactC2-AAA-Ez-AFBD mutant (orange) and re-plated on FN. Scale bar, 10 μm. Note that both Vin-CA and Vin-WT expressing Vin−/− cells have the same anisotropy value on FN indicating full activation of Vin-WT on FN. Dotted magenta lines in A,D outlines the transfected cells. Scale bar 10 μm. All error bars indicate SD. n.s $p > 0.05$, *$p \leq 0.05$, **$p \leq 0.01$, ***$p \leq 0.001$, ****$p \leq 0.0001$. Sample size and p values are provided in Table S4.

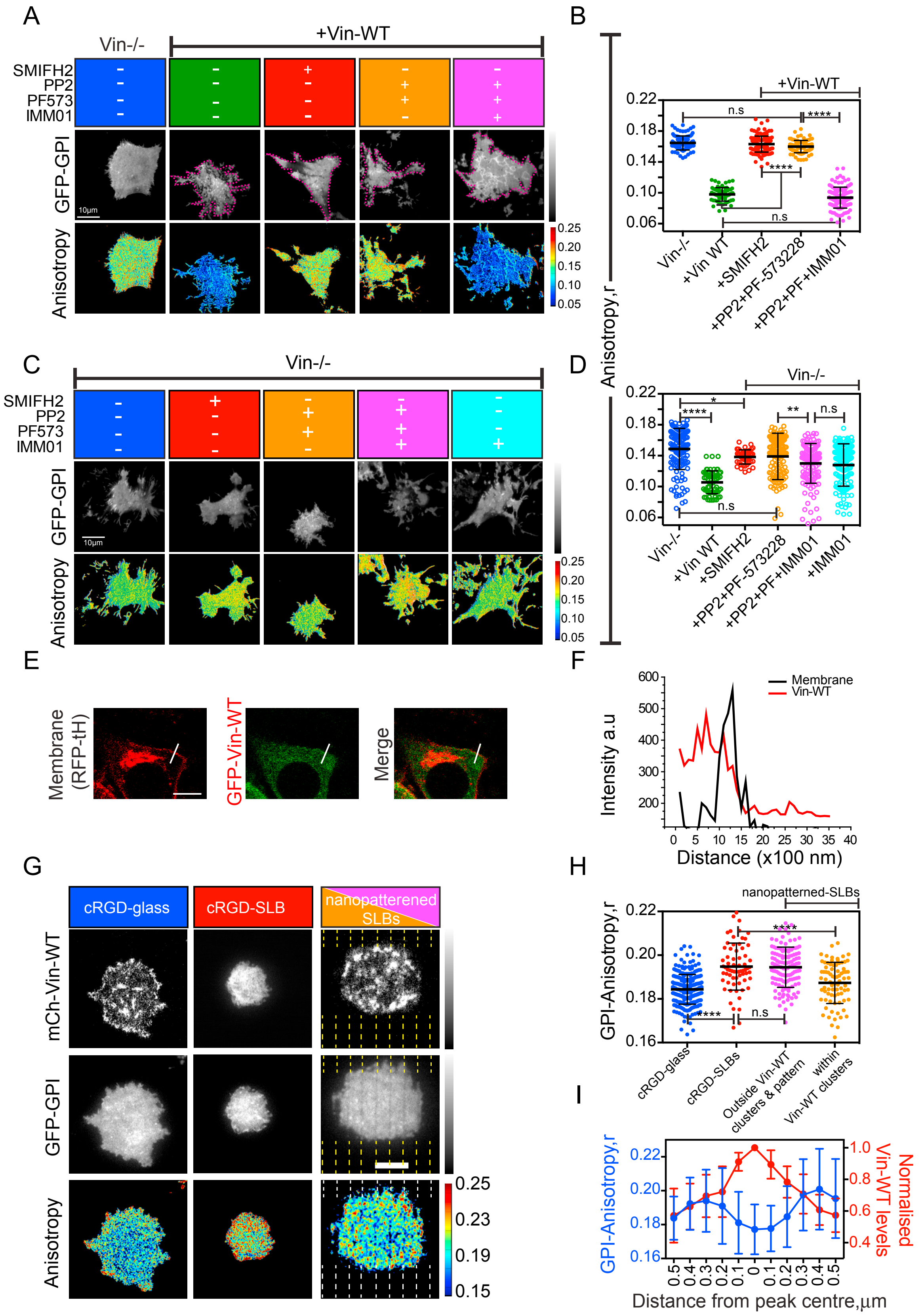

(legend on next page)

Figure S6. Vinculin Functions in the Pathway that Links Actin Activity to Immobilization of Inner-Leaflet Phospholipids and Is Responsible for Localized Generation of GPI-APs in a Force-Dependent Manner, Related to Figure 6

(A-D) Representative intensity and anisotropy images (A,C) and scatter dot plot with mean of ROI anisotropy values (B,D) obtained from GFP-GPI expressing Vin−/− MEFs in the presence (A,B) or absence (C, D) of mCherry-Vinculin WT (+Vin-WT) and pre-treated with the indicated chemical perturbations of the integrin signaling pathway that generates dynamic actin. Note that an increase in GPI-AP anisotropy values was observed only when Vin−/− cells expressing Vin-WT were treated with either the formin inhibitor SMIFH2 (red in A, B) or with an cocktail consisting of both the SFK and FAK inhibitors PP2 and PF573228 respectively (orange in A,B). Vin−/− cells alone do not exhibit significant changes (compare differences between blue and red or orange in C,D with corresponding differences in A,B), upon treatment with the various inhibitors or activators. Also, only in the presence of vinculin does the treatment with the formin agonist IMM01 register a robust change in GPI-AP anisotropy values of PP2 and PF573228 treated cells, indicating a modulation of nanoclustering (compare orange and magenta in A,B with C,D). Treatment of Vin−/− cells with IMM01 (cyan in C,D), does not rescue the nanoclustering defect observed in Vin-/- cells indicating that vinculin operates downstream of the dynamic actin machinery in generating GPI-AP nanoclusters. Dotted magenta lines in A outlines the Vin-WT transfected cells. Scale bar 10 μm. (E,F) Confocal image (E) of Vin−/− MEFs transfected with GFP-tagged Vinculin and co-transfected with a membrane marker (RFP-tHRas) and imaged into the cell in Z. Plot (F) shows the line intensity profiles of GFP-Vin-WT and RFP-tHRas (PM marker) showing the absence of any detectable intensity of Vin-WT at the PM post 12-16 hours of transfection. White line in (E) depicts the region chosen for the line scan measurement. Scale bar 5 μm. (G-I) Representative vinculin channel intensity, GFP-GPI total intensity and anisotropy images (G) of CHO cells transiently transfected with mCherry-Vinculin WT and plated on SLBs with mobile cRGD ligand (red) or on cRGD immobilized on glass via PLL-g-PEG (blue), or on SLBs prepared on nanopatterned surfaces (magenta and orange). The position of the 100nm chromium lines is represented as yellow dashed lines (in intensity images) and white dashed lines (in anisotropy images). Scale bar 5 μm. Scatter dot plot with mean of ROI anisotropy values (H) obtained from GFP-GPI expressing CHO cells co-expressing mCherry-Vin-WT plated under the indicated conditions and comparing anisotropy of GFP-GPI in regions marked by vinculin clusters (orange) or outside segmented clusters and in regions away from grids (magenta). 5-pixel averaged line profiles (I) depicting vinculin intensity (Red) and corresponding GPI-anisotropy (blue) obtained by combining data from multiple ~1 μm line ROIs drawn parallel to the chromium lines and passing through the vinculin cluster-center, after normalizing each vinculin profile by its peak intensity and then combining multiple profiles (n = 10 line profiles) by using the vinculin peaks to align the data. All error bars indicate SD. n.s $p > 0.05$, *$p \leq 0.05$, **$p \leq 0.01$, ***$p \leq 0.001$, ****$p \leq 0.0001$. Sample size and p values are provided in Table S4.

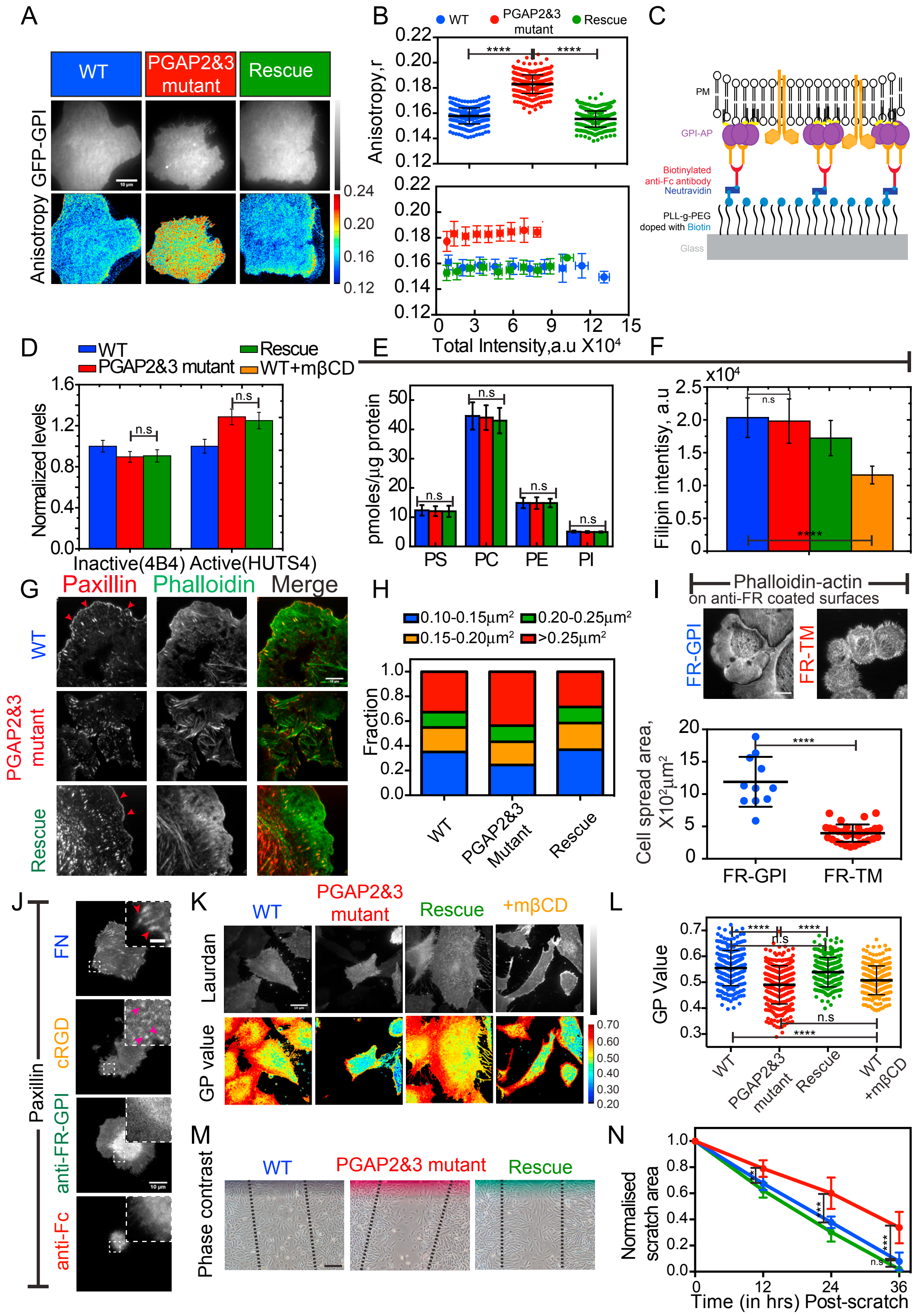

(legend on next page)

Figure S7. Functional Significance of GPI-AP Nanocluster Formation, Related to Figure 7

(A-B) Representative intensity and steady-state anisotropy images (A), scatter dot plot with mean of anisotropy values (B, top) and 2-D scatterplot of mean anisotropy at various total intensity bins (B, bottom) of ROIs obtained from GFP-GPI transiently transfected (12-16 hours) into either wild-type (WT; blue), PGAP2&3 double mutant (red) or PGAP2&3 add back (Rescue;green) CHO cells re-plated onto 10 μg/ml FN coated dishes for 1 hour. Scale bar 10 μm. Note that an increase in anisotropy values in mutant cells was observed corresponding to a loss of nanoclustering of GFP-GPIs. This is rescued by the re-introduction of PGAP2 and PGAP3 enzymes (Rescue cells) and the mutation does not affect GPI-AP expression (B; bottom panel, total intensity axis). (C) Schematic of antibody functionalized PLL-g-PEG-biotin coated glass surfaces. Neutravidin acts as a sandwich layer between PLL-g-PEG-biotin and biotinylated anti-Fc antibody. The anti-Fc antibodies then bind antibodies directed against various cell surface receptors. Cells expressing corresponding epitopes are added to this surface. Additional details can be found in STAR Methods. (D) Quantification of the relative amount of active β1 integrin (marked by HUTS4 antibody) and inactive β1 integrin (marked by 4B4 antibody) (D) normalized to the levels of neutral antibody (K20) in WT cells (blue), PGAP2&3 mutants (red), or PGAP2 and PGAP3 rescued cells (green). Data is also additionally normalized to the levels in the WT. (E,F) MS/MS Mass spectrometric analysis quantifying the mean levels of the indicated phospholipid species (E) and filipin-staining of mean free-cholesterol levels (F) in WT cells (blue) or PGAP2&3 mutants (red) or rescue (green) or WT cells treated with 10mM mβCD (orange; control in F). (G) TIRF images of WT, PGAP2&3 double mutant and Rescue cells, after re-plating on FN coated dishes for 60 mins and subsequently fixed, permeabilized and stained for paxillin (Left panel) to mark focal adhesions, or labeled with Phalloidin to mark actin filaments (Middle panel). The merge of both channels is shown on the Right. Scale bar 10 μm. (H) Bar graph depicting the relative fractions of binned adhesion sizes (marked by paxillin) in WT (first), PGAP2&3 double mutant (middle) and Rescue line (last). Note that the mutant cells have larger adhesions (> 0.25 μm^2) and typically lack the smaller nascent adhesions (< 0.15 μm^2) that are usually found at the periphery/lamellipodia (Red arrow heads in G). (I) TIRF images of Phalloidin stained actin (I;top) of FR-GPI or FR-TM(-Ez-AFBD) expressing CHO cells plated on PLL-g-PEG/PEG-bio functionalized with biotinylated-anti-Fc antibody coupled to anti-FR antibodies. Quantification of cell spread area is shown in the graph (I; bottom). Scale bar, 10 μm. Note the presence of branched actin containing lamellipodia, which largely contributes to the differences in cell, spread area between the FR-GPI and FR-TM expressing cells. (J) FR-GPI cells transiently expressing mApple-Paxillin were plated on either FN (blue), cRGD (orange) or PLL-g-PEG-coated glass functionalized with anti-Fc antibody (red) or additionally with anti-FR antibody (green). Scale bar 10 μm. Inset: zoom of the dashed box. Scale bar in inset 2 μm. Note the presence of focal adhesions marked by paxillin on cells plated on FN (red arrow heads) and small nascent clusters that form on cells plated on cRGD (magenta arrow heads) in the corresponding insets. (K-L) Laurdan fluoresence intensity and generalized polarization (GP) images (K) and scatter dot plot with the mean of ROI GP values (L) obtained from WT cells (blue), PGAP2&3 mutants (red), PGAP2&3 add-back (Rescue;green), or WT cells treated with 10mM mβCD (orange). Scale bar, 10 μm. The lower values of GP observed in the PGAP2&3 mutants indicate the reduction of *lo*-characteristics in the cell membrane similar to that observed when WT cells were treated with mβCD, a well-characterized perturbant of *lo*-domains. Scale bar 10 μm. (M-N) Phase contrast images (M) of mitomycin C-treated WT (blue), PGAP2&3 mutant (red), PGAP2&3 rescued cells (green) imaged 36 hours post-scratch wounding in the location indicated. The dashed black line in each case represents the original position of the scratch. Scale Bar 100 μm (N) Quantification of the mean area of the scratch that was not occupied by cells at the indicated time points normalized to the starting area in each case. All Error bars indicate the SD. n.s $p > 0.05$, *$p \leq 0.05$, **$p \leq 0.01$, ***$p \leq 0.001$, ****$p \leq 0.0001$. Sample size and p values are provided in Table S4.
